# A Complete *Ab Initio* View of Orbach and Raman Spin–Lattice Relaxation in a Dysprosium Coordination Compound

**DOI:** 10.1021/jacs.1c05068

**Published:** 2021-08-16

**Authors:** Matteo Briganti, Fabio Santanni, Lorenzo Tesi, Federico Totti, Roberta Sessoli, Alessandro Lunghi

**Affiliations:** † Department of Chemistry “Ugo Schiff”, INSTM Research Unit, Università degli Studi di Firenze, 50019 Sesto F.no, Italy; ‡ School of Physics, AMBER and CRANN Institute, Trinity College, Dublin 2, Ireland

## Abstract

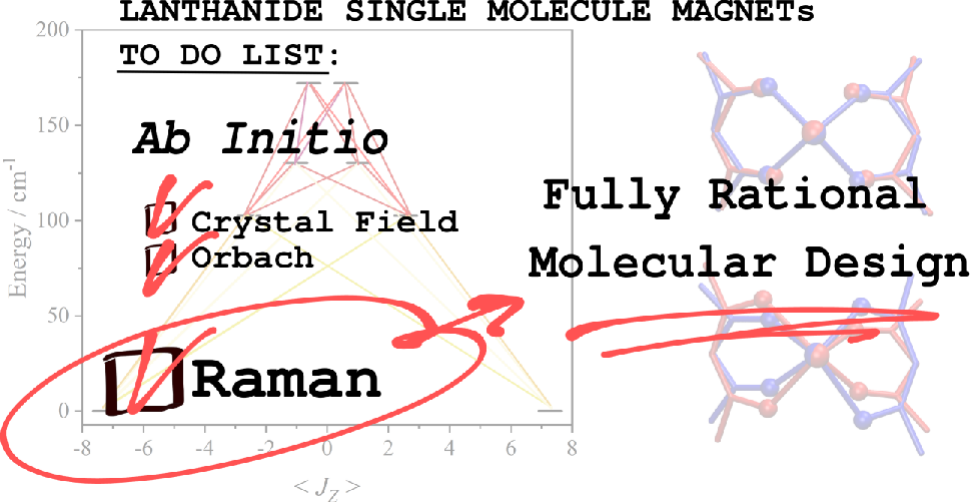

The unique electronic and magnetic properties of lanthanide molecular complexes place them at the forefront of the race toward high-temperature single-molecule magnets and magnetic quantum bits. The design of compounds of this class has so far being almost exclusively driven by static crystal field considerations, with an emphasis on increasing the magnetic anisotropy barrier. Now that this guideline has reached its maximum potential, a deeper understanding of spin-phonon relaxation mechanisms presents itself as key in order to drive synthetic chemistry beyond simple intuition. In this work, we compute relaxation times fully *ab initio* and unveil the nature of all spin-phonon relaxation mechanisms, namely Orbach and Raman pathways, in a prototypical Dy single-molecule magnet. Computational predictions are in agreement with the experimental determination of spin relaxation time and crystal field anisotropy, and show that Raman relaxation, dominating at low temperature, is triggered by low-energy phonons and little affected by further engineering of crystal field axiality. A comprehensive analysis of spin-phonon coupling mechanism reveals that molecular vibrations beyond the ion’s first coordination shell can also assume a prominent role in spin relaxation through an electrostatic polarization effect. Therefore, this work shows the way forward in the field by delivering a novel and complete set of chemically sound design rules tackling every aspect of spin relaxation at any temperature.

## Introduction

Lanthanide elements find widespread use in numerous technological^1^ and biomedical applications.^2^ The partially filled *f*-orbital shell and the large spin–orbit coupling interaction grant these compounds unique magnetic and electronic properties. For instance, they are employed for the mass fabrication of permanent magnets,^3^ while several lanthanide ions are ideal constituents of luminescent probes,^4,5^ sensors,^6^ LEDs,^7^ and MRI contrast agents.^8,9^ Over the last two decades, coordination compounds of trivalent lanthanide ions also acquired a prominent role in molecular magnetism since the discovery that they can show slow relaxation of the magnetization and the opening up of a hysteresis loop,^10−13^ offering a glance at the possibility to create single-molecule magnets (SMMs), namely the molecular version of bulk permanent magnets for high-density data storage.^14,15^ Interestingly, the possibility of obtaining lanthanide complexes with very long magnetic moment lifetimes^16,17^ also makes them optimal candidates for the realization of multilevel quantum bits (qudits).^18−20^

All these applications have one common denominator: their working mechanism stems from a very efficient decoupling of the lanthanide electronic and magnetic degrees of freedom from the atomic motion of the ion’s environment. The latter invariably leads to electronic and spin relaxation and the quenching of the desired properties. In the case of magnetic relaxation of Kramers ions, this decoupling is realized by preventing an electronic state with maximum *z*-component of the total angular momentum, namely *m*_*J*_ = *J*, to evolve into *m*_*J*_ = −*J*. This can be accomplished by a suitable choice of the lanthanide ion’s crystal field in a way that the Kramers doublet (KD) *m*_*J*_ = ± *J* is the ground state and the other states with smaller projection of *m*_*J*_ are well separated in energy and only slightly admixed among them.^21,22^ Under such condition, the transition within the ground-state KD can only be mediated by a sequential number of transitions involving excited KDs and induced by a sequential absorption/emission of one quantum of lattice vibrational energy,^11^ namely a phonon. Ideally, the system would need to climb up to the highest KD. Once the system has reached such a high energy state, it is able to rapidly relax to either sides of the anisotropy barrier by spontaneously emitting phonons. This relaxation mechanism, called the Orbach process, becomes very slow at low temperature due to the lack of thermally available phonons able to initiate the process, and it follows an exponential relation of the type τ = τ_0_ exp(*U*_eff_/*k*_B_*T*), where the pre-exponential factor τ_0_ sets the time scale of relaxation τ and *U*_eff_ is the anisotropy barrier that the magnetic moment needs to overcome in order to invert its orientation. This temperature dependence has been successfully used to reproduce the behavior of 3*d* SMMs such as Mn_12_, Fe_8_, etc.,^23−25^ whose spin levels span an energy range smaller than the energy of the first optical vibrational modes. The increase of *U*_eff_ has been at the center of the synthetic efforts in the field and has led to the design of molecular complexes with anisotropy barriers exceeding 2000 K.^26−28^

Although very effective synthetic strategies have pushed *U*_eff_ to its maximum potential value,^29^ their success is undercut by two adverse effects: the small value of τ_0_, as short as 10^–12^ s,^28,30^ and the rise of an additional relaxation mechanism at low temperature, generally referred to as Raman^11^ on the basis of phenomenological models.

Until now, a clear chemical interpretation of τ_0_ and Raman relaxation in terms of molecular motion has not been available for lanthanide complexes. Further progress in this field is only possible through understanding of the physical nature of spin-phonon relaxation by an in-depth first-principles description of how the solid-state molecular environment affects the spin degrees of freedom. Only in the past few years significant advances have been achieved with the introduction of *ab initio* methods for the prediction of spin-phonon coupling^31−33^ and spin-phonon relaxation time.^26,34^ On the basis of these seminal contributions, both transition-metal-based spin qubits^35−38^ and mononuclear single-molecule magnets^33,34,39−44^ have been studied to disentangle the influence of the spin-vibrational coupling and of the effective phonon density of states (DOS) on the temperature dependence of relaxation rates. The community has recently showed a large interest in simulating relaxation time in lanthanide complexes, including second-order mechanisms responsible for Raman relaxation,^45^ but so far investigations have either included phenomenological parameters^45^ or have been limited to computing vibrations at the gas-phase molecular level.^26,28,40,46^ Such approaches, although insightful, risk to hide important details of spin dynamics or do not take into account the lower part of the phonons spectrum. The latter has indeed been proposed to be crucial to understand low-temperature relaxation mechanisms.^34,41,42,45^

In this paper, we make a significant step forward by providing the first accurate and fully *ab initio* description of the one- and two-phonon processes that lead to magnetic relaxation in a prototypical lanthanide SMM. The success of a fully *ab initio* theory of magnetic relaxation is pivotal toward the unambiguous interpretation of the relaxation mechanisms and provides the stepping stone for a detailed analysis of the origin of relaxation itself. Backed up by a rigorous theoretical and computational framework, it is now possible to go beyond simple arguments based on phenomenological models, look at the molecular origin of spin-phonon coupling, and propose a comprehensive chemical strategy against fast spin-phonon relaxation.

The molecule selected for our study is [Dy(acac)_3_(H_2_O)_2_]·EtOH·H_2_O compound^47^ (acac^–^ = acetylacetonate, EtOH = ethanol), **Dyacac** for short. This molecule was among the first single-ion lanthanide complexes to have been reported to show slow relaxation of its magnetic moment in absence of a static external magnetic field, and its magnetic relaxation behavior represents a fingerprint for most of the many lanthanide SMMs reported in the literature. An additional factor in favor of **Dyacac** is that this compound is simple enough to permit a detailed analysis and, at the same time, it allows a fully *ab initio* investigation without prohibitive computational requirements.

Computational predictions of spin-relaxation time compare well with susceptometry measurements and allow us to demonstrate that second-order Raman relaxation is indeed an excellent candidate to explain the low-temperature relaxation regime. Moreover, we perform a detailed investigation on how spin-phonon coupling emerges from molecular motion, thus providing a chemical interpretation of both Orbach and Raman relaxation. Surprisingly, our results show remarkably large contributions from atoms beyond the first coordination shell as a result of electrostatic polarization. This finding defies the intuition that 4*f* magnetic states, being strongly localized, are only affected by distortions of the first coordination shell and points toward ligands with donor atoms weakly polarizable by intramolecular vibrations as a new chemical strategy to reduce spin-phonon coupling.

## Results

### The Dy^3+^ Crystal Field: Static *Ab Initio* Calculations and Validation by Cantilever Torque Magnetometry

In the investigated compound, the Dysprosium atom is coordinated by three acac^–^ ligands and two water molecules, with a total coordination number of eight. Although the geometry around the lanthanide ion can be approximated to a structure with point-group symmetry *D*2*d*, the actual symmetry is too low to provide hints about the orientation and the values of the components of the magnetic anisotropy tensor. *Ab initio* simulations and cantilever magnetometry are used to infer the electronic structure of **Dyacac**.

In order to accurately simulate the chemical environment of the Dy ion, our model includes (i) the molecular unit made of [Dy(acac)_3_(H_2_O)_2_] and two cocrystallized ethanol and water molecules and (ii) a surrounding 7 × 5 × 7 supercell of point charges placed at the atomic crystallographic positions to reproduce the Madelung potential inside the crystal. The crystal structure and cell parameters are optimized by periodic density functional theory (pDFT) methods, while atomic point charges, computed by pDFT on the optimized crystal structure, are employed (see Computational Methods, section S1.1 in the Supporting Information). The optimal size of the point charges’ supercell is evaluated by computing the electronic structure as a function of the number of charges (see section S2.1 in the Supporting Information). All the calculations concerning magnetic properties are performed at the Complete Active Space Self Consistent Field (CASSCF) level of theory, followed by Complete Active Space State Interaction by Spin–Orbit interaction (CASSI-SO) (see the Computational Methods in the Supporting Information for further details, sections S1.1 and S1.2).

**Figure 1:**
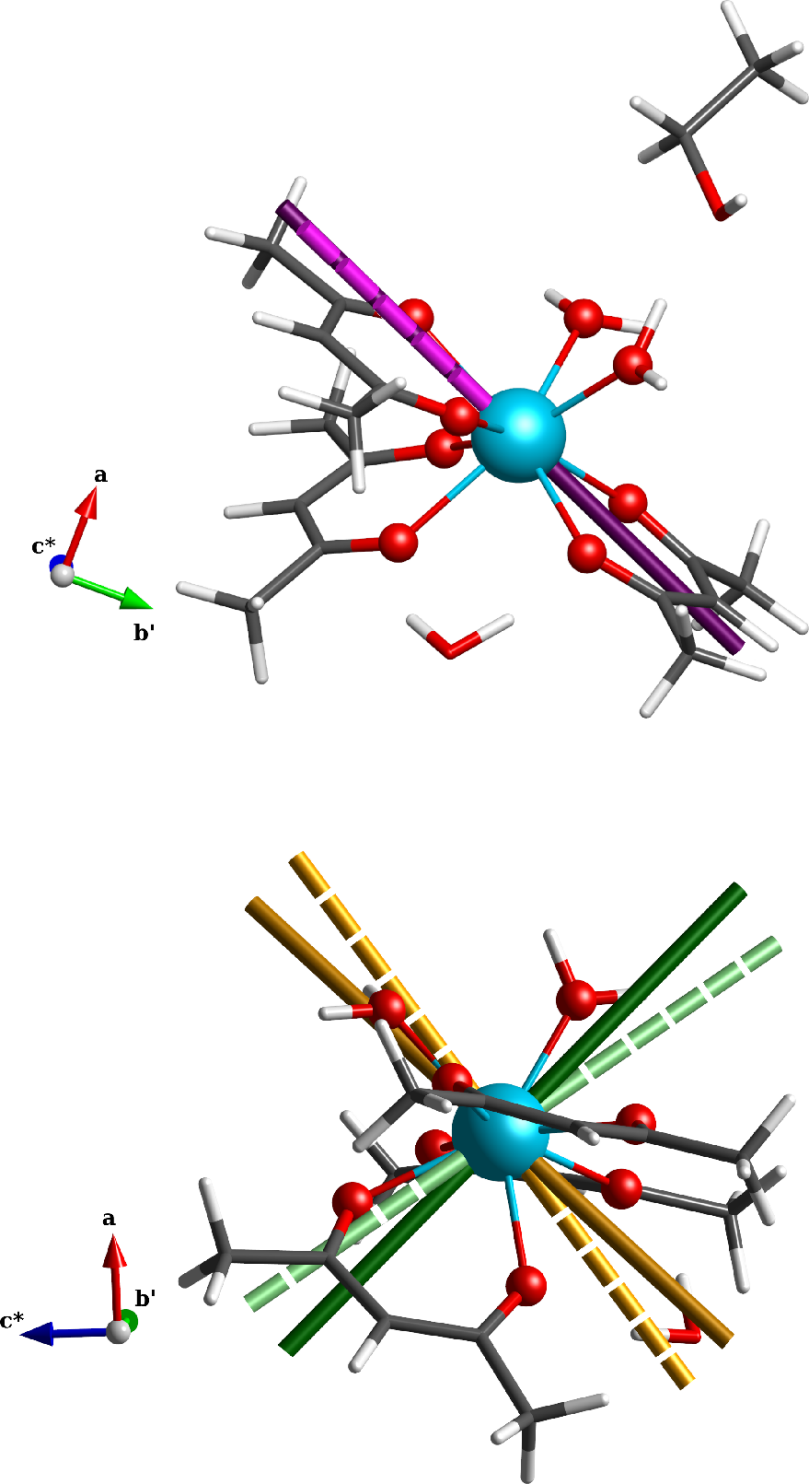
Magnetization principal axes. The molecular structure of **Dyacac** together with the principal anisotropy axes of ground KD as obtained by simulation with the *ab initio* parameters (dashed axes and light colors) and by fit of CTM experimental curves (continuous axes and dark colors). Top and bottom panels show different molecule orientations to highlight the different axes: easy axis for top, intermediate, and hard for bottom. Axis color code: magenta = easy axis, orange = hard axis, and green = intermediate axis. Atomic color code: aquamarine = Dy, red = oxygen, gray = carbon, white = hydrogen.

Despite the lack of symmetry, the computed **g**-tensor of the ground KD is quite axial (*g*_*X*_ = 0.045, *g*_*Y*_ = 0.089, *g*_*Z*_ = 19.371, see Table S6), a common feature for Dy^3+^ complexes despite the low symmetry coordination geometries, as recently pointed out and rationalized.^48^ The direction of the easy axis (see Figure 1) is almost parallel to the bond with the closest oxygen atom of one acac^–^ ligand, as commonly found.^49,50^ Such a direction can be rationalized in the following way: in order to minimize the electrostatic repulsion with the ligands, the oblate electron density of the ground *m*_*J*_ = ± 15/2 doublet is mostly localized in the plane defined by the two water molecules’ oxygens (neutral), which also present the longest bond lengths (see Figure 1).

The computed electronic structure shows the first and second excited doublets lying at 101 and 129 cm^–1^, respectively, and with the whole  multiplet spanning 450 cm^–1^. Such values are quite common for Dy complexes even in low-symmetry environments, as demonstrated by the many examples existing in literature.^11^ The accuracy of *ab initio* calculations is validated by comparison with the experimental magnetic anisotropy determined by Cantilever Torque Magnetometry (CTM).^51−56^ In particular, CTM on single crystals is a very sensitive tool allowing measurements over a wide temperature range, thus probing also excited spin states.^57−59^

**Figure 2:**
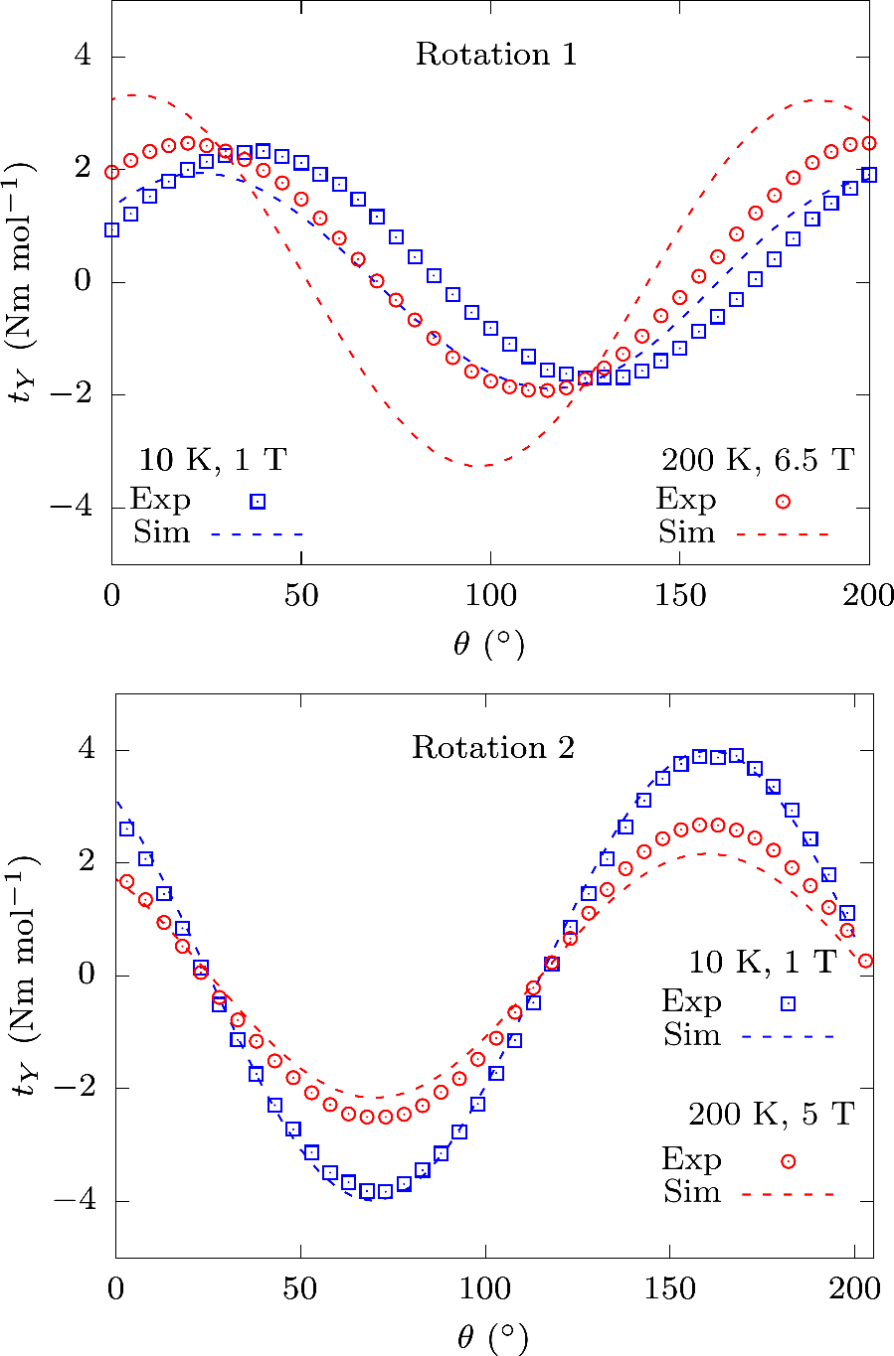
Magnetization torque measurements. The torque momentum measured on the **Dyacac** single crystal as a function of the rotation angle at 10 K (blue square symbols) and 200 K (red circle symbols) for Rot1 (top panel) and Rot2 (bottom panel). The simulations (broken lines) are based on the parameters obtained by *ab initio* calculations. (See the Experimental Methods in the Supporting Information.)

In the experiment, a freshly prepared **Dyacac** single crystal (see the Experimental Methods in the Supporting Information, sections S1.5 and 1.6) was indexed by X-ray diffraction and placed on a rotating cantilever, which was inserted into the cryostat. Two sets of orthogonal rotations (Rot1 and Rot2) were performed under an external magnetic field, *B⃗*. The measured component of torque momentum *t⃗* is given by the equation
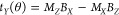
1where *XYZ* is the laboratory reference frame, θ the rotation angle, and *M⃗* the sample magnetization. By rotating the sample, it is possible to map the projection of the magnetic principal axes on a defined plane, and every time one of these is parallel to *B⃗* the torque is zero. Being the orientation of the crystallographic frame, abc*, known with respect of the laboratory reference frame, it is possible to identify the directions of the principal magnetic anisotropy axes in the molecular frame, *xyz* (see the Experimental Methods in the Supporting Information, sections S1.5 and 1.6). The results of the CTM experiments for the two rotations obtained for the lowest and highest investigated temperature values are shown in Figure 2, while all results are displayed in the Supporting Information, section S3.

*Ab initio* calculations provide all information necessary to simulate the CTM experimental results, i.e., the parameters *B*_*m*_^*l*^ of the crystal field Hamiltonian
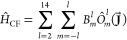
2where *Ô*_*m*_^*l*^(**J⃗**) are tesseral tensor operators.^60^ These quantities allow us to calculate magnetization in all directions of space and the magnetic anisotropy tensor at all temperatures. The comparison between experimental results and simulation is reported in Figure 2. For Rot1, the simulation reproduces the experimental trend, despite a marked offset of ca. 14°, and it overestimates the torque at high temperature. On the other hand, Rot2 exhibits a striking agreement between simulations and experiments even up to 200 K. The principal ground KD anisotropy axes obtained by *ab initio* calculations can be visualized in Figure 1 and Figures S19 and S20. A refinement of the magnetic anisotropy orientation can be done by fitting the torque experimental data. However, only minor variations in the main magnetic axes’ orientations are observed, particularly regarding the magnetic easy axis (see the Experimental Details in the Supporting Information, section S3 and Figure S18). From this analysis, the high reliability of *ab initio* calculations emerges, despite a minor tilt of the hard directions that are more difficult to reproduce.^61−63^

### Spin–Lattice Relaxation

The Crystal Field Hamiltonian of eq 2 describes the energy levels of the ground-state electronic multiplet for the equilibrium molecular geometry. However, at finite temperature, the position of the atoms inside the lattice fluctuates due to thermal energy. These structural fluctuations are the origin of spin-phonon coupling and have the ability to elicit transitions between different electronic states until the electronic energy levels reach the thermal equilibrium with the lattice. This phenomenon is captured by two sets of quantities, the vibrational modes of the lattice, *q*_α_, and the spin-phonon coupling operator , which describes the effects of the α-lattice vibration on *Ĥ*_CF_. All these parameters can be calculated from first-principles following a strategy outlined on a series of previous works by some of the authors^31,34^ and extensively benchmarked on transition metal ion qubits^35,36,64^ and single-ion magnets.^41^ Details of all calculations are reported in the Methods section in the Supporting Information, sections S1.3 and S1.4. In a nutshell, phonons are calculated from pDFT employing the crystal unit cell. Unit cell contains four molecular units and several interstitial water and ethanol molecules, for a total of 244 atoms. Vibrations computed on the periodic crystal’s unit cell include low-frequency lattice distortions, where intramolecular motion is admixed to rigid molecular translations and rotations. Including all the phonons at the Γ-point is crucial to fully capture the relaxation behavior of these compounds, and it is the first fundamental step toward a full integration of the Brillouin zone as attempted elsewhere.^41^ Spin-phonon coupling coefficients are instead calculated by numerically differentiating the CF parameters by computing the values of *B*_*m*_^*l*^ along many molecular distortions with the CASSCF method. Once all these quantities have been computed, it is possible to predict the transition probability due to absorption or emission of phonons through a formalism very similar to the Fermi Golden Rule employed to predict spectroscopic transitions. In this work, we consider first- and second-order time-dependent perturbation theory, where spin-phonon coupling, *V̂*_α_, is the perturbation and *Ĥ*_CF_ of eq 2 is the unperturbed Hamiltonian. In this framework, it is possible to compute both one- and two-phonon processes. In the nomenclature of spin–lattice relaxation, these processes correspond to the Orbach and Raman relaxation mechanism, respectively. The former relaxation mechanism involves the transition between two spin states *a* and *b* due to the absorption or emission of a single phonon *q*_α_. The rate of this process is described by eq 3
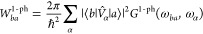
3where *G*^1-ph^ = δ(ω – ω_α_)*n̅*_α_ + δ(ω + ω_α_)(*n̅*_α_ + 1) and *n̅*_α_ = [exp(*ℏω*_α_/*k*_B_*T*) – 1]^−1^ is the Bose–Einstein distribution of thermal population, *ℏω*_α_ is the α-phonon energy and *k*_B_ is the Boltzmann constant. A similar but more rigorous expression for one-phonon process that includes the contribution of spin-states coherence was previously derived on the basis of the Redfield formalism^35^ (see the Methods section in the Supporting Information, section S1.4) and it is here used for the computation of one-phonon contributions to relaxation time.

The Raman relaxation mechanism accounts for transitions among two spin levels *a* and *b*, and it is mediated by the simultaneous absorption and/or emission of two phonons and a contribution of all the electronic excited states. The Raman rate is modeled with the expression
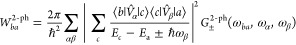
4where *G*_±_^2-ph^, reported in full in the Methods section in the Supporting Information, accounts for the thermal populations of phonons and imposes the conservation of energy, analogously to *G*^1-ph^ for the Orbach process. There are three possible processes involving two phonons: absorption of two phonons, emission of two phonons, and simultaneous emission of one phonon and absorption of another one. For instance, the emission of a phonon *q*_α_ and the absorption of a phonon *q*_β_ contribute to *G*_±_^2-ph^ as
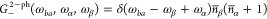
5

The Raman process connecting two spin states *a* and *b* also involves a contribution from all of the other spin states *c*. The contribution of this envelope of intermediate excited states, often referred to as a virtual state, does not involve any intermediate real transition to any of those states *c*, as it instead happens for a series of one-phonon processes in the Orbach relaxation. On the contrary, it only represents the fact that the spin states are no longer eigenstates of the total system (spin plus phonons) and that in such a condition all the KDs get slightly admixed among them by the external perturbation, i.e., the phonons.

All parameters in eqs 3 and 4 are computed fully *ab initio* with the sole exception of a Gaussian smearing used to approximating the Dirac delta function. The small dependency of the results on this parameter is discussed in the Supporting Information, section S2.5. We also note that this degree of freedom can also be eliminated by further including the integration of the phonons across the Brillouin zone.^35,41^ The relaxation time τ for the Orbach and Raman relaxation is computed as the second-smallest eigenvalue of the matrices *W*^1-ph^ and *W*^2-ph^, and it is reported in Figure 4 for different values of temperature.^24^

**Figure 3:**
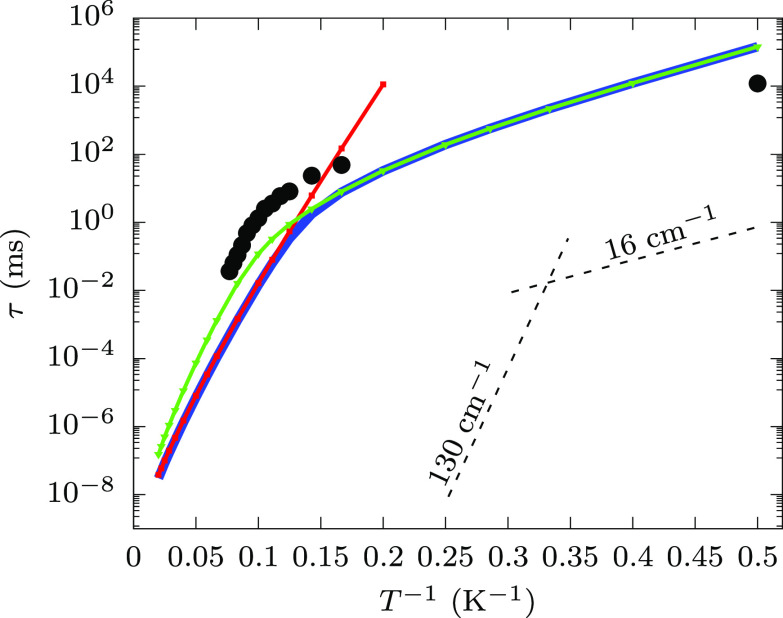
Temperature dependence of the spin-phonon relaxation time. The continuous blue line reports the total computed relaxation time coming from both the Orbach and Raman mechanism. The former contribution is reported explicitly with a red line, while the latter is reported with a green line. Solid symbols of the same colors indicate the temperatures at which simulations were performed. The black circles report the experimental relaxation times obtained from AC susceptibility measurements in the absence of external field, for 6 K ≤ *T* ≤ 13 K, and from hysterisis loop at 1500 Oe external field, for *T* = 2 K. Dashed black lines corresponding to exp(*U*_eff_/*k*_*B*_*T*), with *U*_eff_ = 130 and 16 cm^–1^ are reported in the bottom right part of the plot as a guide to the eye to interpret results.

**Figure 4:**
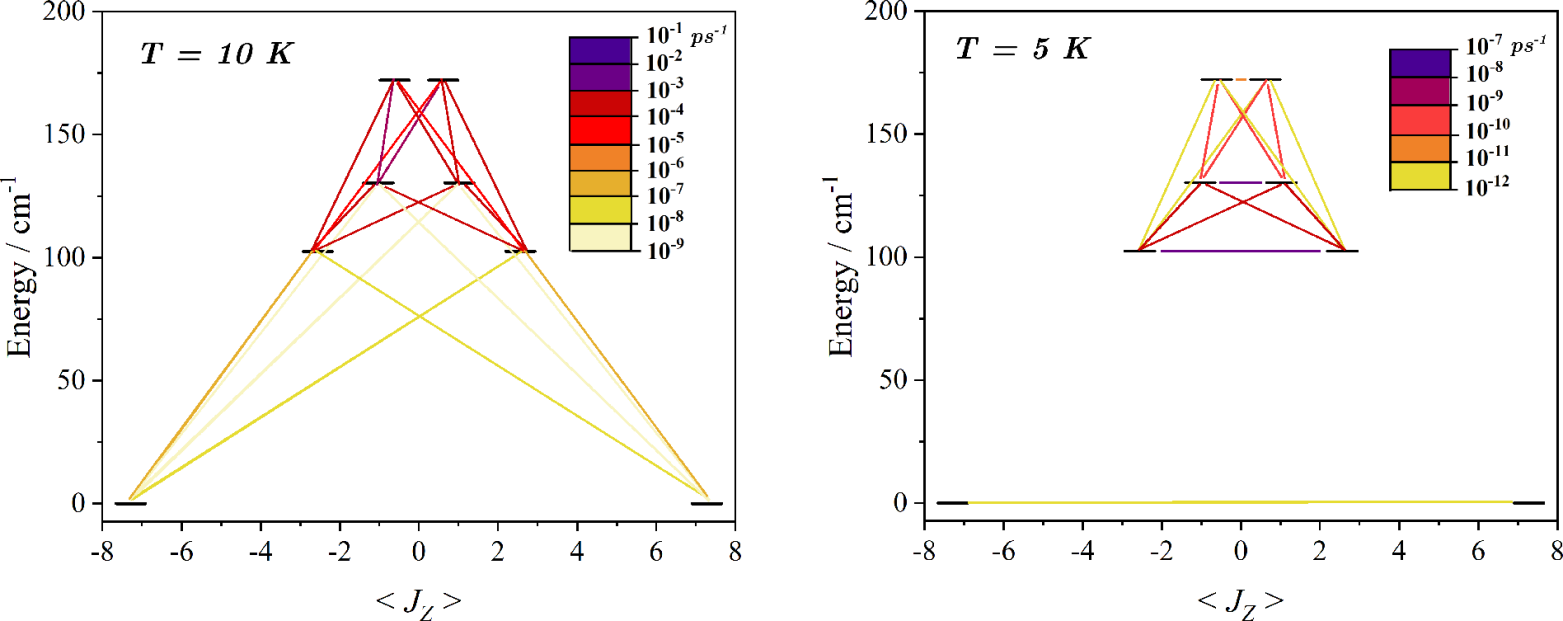
Finite temperature Orbach and Raman computed transition rates. Right and left panel report the matrix elements of *W*^1–*ph*^ and *W*^2–*ph*^, respectively, expressed in the basis of the eigenvectors of the ground KD’s **g**-tensor. On the *x*-axis, it is reported the computed average magnetic moment for the first four KDs and their energy separation from the ground state. All the rates are expressed in ps^–1^ and always refer to absorption rates. The emission rates are always found to be much larger because of the spontaneous emission contribution.

The Orbach and Raman mechanisms are predicted to dominate relaxation at high and low temperature, respectively. The value of *U*_eff_ associated with the Orbach mechanism is generally extracted by fitting the slope of the linear relation of ln(τ) vs (*k*_B_*T*)^−1^. Simulations show that a perfect linearity is absent, but by focusing on the high-temperature data it is possible to identify a slope of *U*_eff_ ∼ 130 cm^–1^. This value coincides with the energy of the second excited KDs. This is in agreement with the expression for the Orbach mechanism of eq 3, where phonons in resonance with the electronic transitions are more efficiently absorbed or emitted. However, the nonperfect linearity of ln(τ) vs (*k*_B_*T*)^−1^ suggests that the relaxation is mediated by different KDs at different temperatures, with higher excited ones taking over relaxation as temperature increases and resonant phonons become populated. On the other hand, the Raman mechanism is computed to dominate relaxation at low temperature, with the relaxation time following a power-law with respect to temperature up to ∼8 K.

To get a deeper insight into the temperature dependence of the relaxation time, we analyzed the transition rates for the two mechanisms. The left panel of Figure 4 describes the computed transition rates for the Orbach mechanism at a temperature of 10 K. It should be noted that such transition rates are not computed as the expectation value of the magnetic dipole moment, as often reported in literature,^65,66^ but instead are computed from the true *ab initio* spin-phonon transition rates used to determine τ itself. Figure 4 highlights that an intraground KD transition has zero probability, as a consequence of the Kramers theorem, but the interwell transition from the ground state KD to the first and second excited KDs is very likely and offers an efficient relaxation pathway. The transition rates of the Raman relaxation at 5 K are reported in the right panel of Figure 4 and show a reverse situation with respect to the Orbach case. At the second order of perturbation theory, the intra-ground-state KD transition is possible and dominates the relaxation mechanism. This is made possible by the presence of the excited KDs, which allows the simultaneous absorption and emission of two phonons. Interwell transitions among the excited states are also promoted by the Raman mechanism but have a negligible role at low temperatures because these levels are not populated.

Equation 4 shows that at the second order of perturbation theory, among all the phonons in the spectrum, those contributing to relaxation are those best satisfying three requirements: (i) the absorbed phonon is more thermally populated than others, (ii) its energy must be close to the transition energy of the excited KDs, and (iii) the energy conservation condition contained in *G*^2-ph^ requires that the sum of the energy of absorbed and emitted phonons matches the energy difference between the initial and final spin state, i.e. the single phonons do not need to be resonant with the spin transitions. In the case of a Kramers system in zero external field, condition (iii) implies that the absorbed and emitted phonons must be degenerate. Considering Raman relaxation at 5 K, conditions (i) and (ii) are in antithesis, as only phonons with much lower energy of the first excited KD, computed at 100 cm^–1^, will be significantly populated. Which of the two requirements is more stringent can be deducted by the fact that phonons’ thermal population diminishes exponentially with their energy, as enforced by the thermal population term present in eq 5, while requirement (ii) is only enforced to the power of two, as evinced by the denominator of the relaxation rate prefactor in eq 4. Therefore, the best compromise of conditions (i)–(iii) at low temperature is fulfilled by the absorption and emission of a degenerate pair of the first available optical modes, here computed to start at ∼16 cm^–1^ (see also *G*^2-ph^ in Figure S5). In such a normal mode, the acac-ligands’ internal degrees of freedom and the Dy–O bonds remain mostly rigid, while the angles between the Dy ion and the planes containing the acac’s π systems are the only structural parameters significantly affected during the vibration (see Figure S4 and animated videos in the Supporting Information).

Thus, for the Raman relaxation that directly connects the two sides of the anisotropy barrier in the regime of *ℏω*_α_ > *k*_B_*T*, two degenerate phonons of this lowest in energy normal mode contribute to the *T* dependence of τ^–1^ through *G*^2-ph^ = *n̅*_α_(*n̅*_α_ + 1) ∼ exp(−*βℏω*_α_). Such a contribution would appear as straight line in the ln(τ) vs 1/*T* plot of Figure 3, with slope of ∼16 cm^–1^. However, several pairs of phonons become more and more populated for increasing values of *T*, therefore adding their contribution to τ. This translates into a power-law relation of τ vs *T* in the low-*T* Raman-dominated relaxation regime.

Now that we have fully analyzed the computational results, we turn to their comparison with experimental ones. The magnetization dynamics of this compound was previously investigated by Jiang et al.^47^ However, given the tendency of the crystals to lose ethanol crystallization molecules, the characterization was repeated using a freshly synthesized **Dy_0.1_acac** sample (90*%* diamagnetically Y^3+^ isostructural analogous) removed from the mother liquors immediately before the measurements (more details are available in the Methods in the Supporting Information, section S1.7). The relaxation times as a function of the inverse of temperature, shown in Figure 3, compare well with the previous results. Below 6 K, the maxima of imaginary component of susceptibility fall at too low frequencies to be detected, but the relaxation time is still too short to be estimated by the time decay of the magnetization. To acquire data on a larger temperature range, we extracted the relaxation time of magnetization by hysteresis measurements performed at 2 K (see the Methods in the Supporting Information, section S1.7). As becomes clear by comparing experiments and simulations, the range of temperatures scanned experimentally falls exactly in the regime where the Raman mechanism, dominating at low temperature, gives way to the Orbach one, which drives spin relaxation at high temperature. Besides being able to semiquantitatively predict relaxation time, our simulation allows for an unambiguous determination of the underlying mechanism of relaxation.

Comparing the agreement between computed and measured τ, the former are around two orders of magnitude shorter. This discrepancy is in line with the state-of-the-art in the field,^41,46^ and it is probably due to deficiencies in the prediction of the many *ab initio* parameters used to compute the relaxation time. For instance, the comparison of measured and predicted CTM shows only semiquantitative agreement, suggesting that a more accurate determination of the lanthanide’s CF might improve the agreement between relaxation times. Similarly, phonons calculations are known to be affected by small inaccuracies due to the lack of anharmonic shifts or deficiencies in the description of the lattice’s dispersion forces from DFT theory. Vibrational shifts in the order of 1–10 cm^–1^ are generally observed between simulations and experiments,^33,37,45,64,67,68^ and a careful benchmark of these methods is a mandatory next step for the advancement of the field. Unfortunately, a direct comparison with experimental THz absorption spectra was not possible because the compound loses solvent molecules of crystallization when the sample space is pumped at room temperature to remove water.

Contrary to what is observed at high temperature, the computed relaxation time at 2 K is slightly longer than the experimental one. However, we would expect to recover the same trend between simulations and experimental values observed at higher temperature, if it was possible to completely remove the contribution of dipolar cross-relaxation in experimental relaxation rates and by adding the contribution of acoustic phonons to calculations. It has been shown for a Co^2+^ SMM that the latter approximation does not affect dramatically the results but might slightly reduce spin lifetime at very low temperature, where only the acoustic phonons are significantly populated.^41^

Interestingly, the computed temperature dependence of τ due to the Raman process described here cannot be easily disentangled from the Orbach mechanism in the presence of anharmonic phonons.^34,36^ A correct estimation of anharmonic interactions will require the computation of linewidths from first-principles and perturbation theory.^69^ Such a calculation is beyond the reach of current simulations, and we postpone the pursuit of such a challenge to future work. However, we notice that some authors^46,70^ have recently reported that anharmonicity is unable to reproduce the experimental temperature dependence of τ. As discussed in the Supporting Information, section S2.6, such a discrepancy from our findings results from the assumption of a Gaussian smearing in place of the physically correct Lorentzian one proposed in ref (34), together with the lack of a proper integration of the vibrational Brillouin zone.^35,36,41^

### The Role of Molecular Vibrations and Electrostatic Polarization

**Figure 5:**
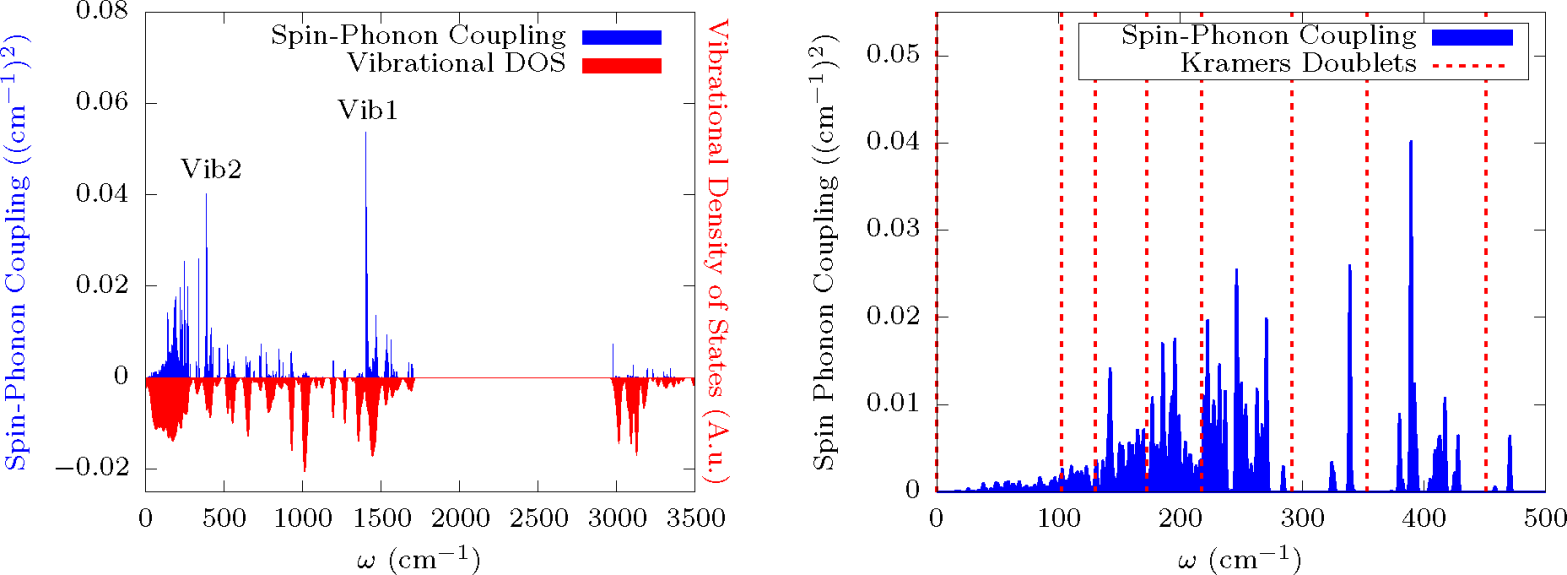
Spin-phonon coupling distribution and vibrational density of states. The left panel reports the spin-phonon coupling distribution and the vibrational density of states as a function of energy. A Gaussian smearing with σ = 1 cm^–1^ and σ = 10 cm^–1^ has been applied to the two functions, respectively. The right panel is a close-up of the spin-phonon coupling distribution overlapped to the energy resonance of the KDs.

Having identified how one- and two-phonon mechanisms affect the temperature dependence of the relaxation time, we can now turn to the analysis of spin-phonon coupling and how it is influenced by the chemical structure and composition of **Dyacac**’s lattice. The left panel of Figure 5 reports the average spin-phonon coupling coefficients as a function of the phonon’s frequency, together with the vibrational DOS. The right panel of Figure 5 instead shows in more detail the spin-phonon coupling in the spectral region that overlaps with the KDs energies. No strict correlation between the spin-phonon coupling intensity and the vibrational density of states emerges, and this is a consequence of the dependence of spin-phonon coupling strength on the nature of the specific vibration. For instance, the spectral region around 1000 cm^–1^ is significantly populated by vibrations but they do not show any significant coupling to the spin. A visual inspection of these modes reveals that the coordination sphere of **Dyacac** is only slightly involved in these vibrations, which are instead strongly localized on the acetilacetonate’s methyl groups rotations or entirely on other molecules, such as on the EtOH molecules. The same effect is observed for the lowest part of the spectrum. Starting from zero energy, spin-phonon coupling increases at a much slower rate than the vibrational density of states. This is due to the fact that the lowest energy modes are dominated by rigid translations of the molecules in the lattice. This kind of motions are not in principle coupled to spin, but their slight admixing with intramolecular distortions can lead to a finite coupling.^31,35^ Such an admixture (Figure 5, right panel) increases as the energy of vibrations approaches the typical values of intramolecular modes in the THz spectral region.

**Figure 6:**
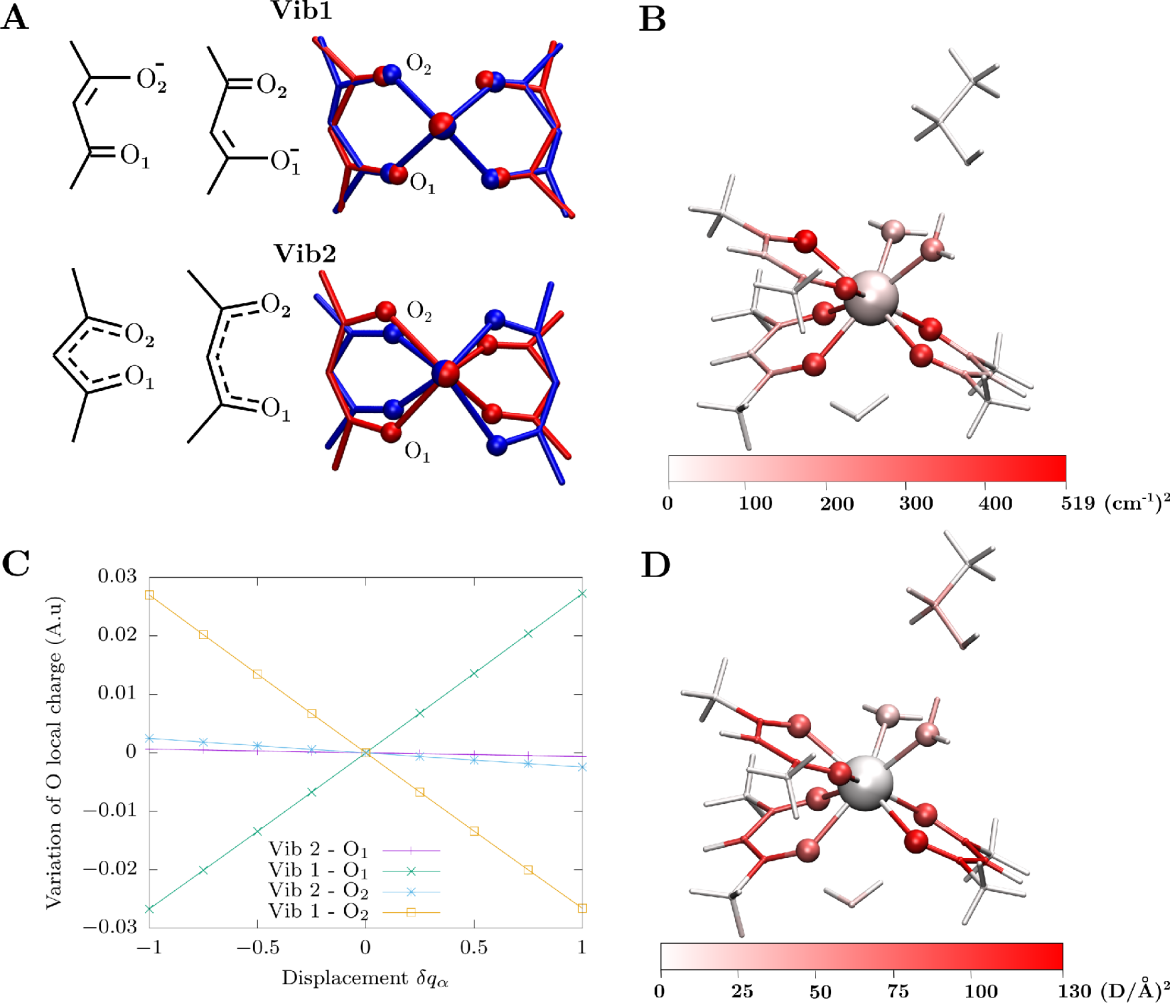
Electrostatic contributions to spin-phonon coupling. (A) Schematic representation of **vib1** and **vib2**. For the sake of clarity, we show only the two acac ligands where most parts of the normal modes are localized (acac1 and acac2 in Figure S1). (B) Magnitude of the spin-phonon coupling coefficients resolved by atomic site. (C) Variation of computed Loprop atomic charge on O_1_ and O_2_ as a function of the displacement along the normal coordinates for **vib1** and **vib2**. (D) Magnitude of the first derivative of electric dipole resolved by atomic site. Module of the derivative for Dy has been set to zero (see the SI) to highlight the values’ variations in the organic scaffold.

Based on these observations and previous studies, one would be tempted to conclude that the largest spin-phonon coupling is triggered by molecular motions strongly distorting the first coordination shell of Dy^3+^. Although not wrong, the analysis of **Dyacac**’s spin-phonon coupling reveals that this is not the entire picture. We now perform a detailed analysis for two modes among the most strongly coupled ones: **vib1** and **vib2**, with resonances at ∼1500 and ∼380 cm^–1^, respectively. Both modes are labeled in the left panel of Figure 5, and they are pictorially shown in Figure 6A (also see the animated videos in the Supporting Information). The choice of these two modes resides on the fact that they show a very high coupling and at the same time present rather different features in terms of atomic displacements. **vib2** is characterized by a significant distortion of the first coordination shell, by breathing of the Dy-acac chelate rings, while CO bond lengths remain quite rigid. **vib1** is largely characterized by a stretching of all the acac ligand’s carbonyl groups, with Dy–O distances being less affected. **vib1**, differently from **vib2**, is expected to significantly affects the acac’s conjugated system by localizing the π electrons toward the two Lewis limit configurations. We note that these vibrations mostly involve two acac ligands, leaving the third one and the water molecules almost unaffected. Although there are other vibrations of similar nature but affecting other pairs of ligands, the choice of **vib1** and **vib2** does not limit the generality of our discussion.

The nature of **vib1** suggests that atoms beyond the first coordination shell might play a significant role in inducing relaxation. However, normal modes are always delocalized on the entire molecules to some degrees, and even **vib1** presents some distortions of the distances and angles between the Dy^3+^ ion and oxygen atoms. We computed the magnitude of the spin-phonon coupling coefficients resolved by atomic site, as presented before^31^ and defined in the Methods sections in the Supporting Information, section S1.3. The results are plotted in Figure 6B and show a remarkable finding: the *sp*^2^ carbon atoms belonging to the acac ligands, namely the Dy’s third nearest neighbors, are found to be coupled to spin at least as much as the oxygen atoms of the waters directly bound to Dy. In order to understand the origin of this phenomenon we perform a detailed analysis of the role of covalent and electrostatic interaction on spin-phonon coupling.

For both **vib1** and **vib2**, we compute an atomically resolved expansion of the electrostatic potential for different vibrational amplitudes and compute the crystal field parameters by replacing one acac ligand (acac1 in Figure S1) with the corresponding atomic point charges, dipoles, and quadrupoles within the LOPROP scheme.^71^ Such an electrostatic analysis allows us to extrapolate atomic-centered multipolar expansion from the computed all-electron CASSCF wave function.^72,73^ The spin-phonon coupling coefficients obtained with an electrostatic model that includes only the contribution of the acac’s oxygen donor atoms are in excellent agreement with the original ones obtained with the explicit model. This demonstrates that, although the static crystal field is generally determined by both electrostatic^21^ and covalent interactions,^74^ its modulation by small atomic displacements, i.e., the spin-phonon coupling, is largely driven by the electrostatic effects of **Dyacac**’s first coordination sphere.

In order to reconcile the observation of a strong effect of Dy’s second and third nearest neighbors and a spin-phonon coupling dominated by the sole electrostatic contribution of the first coordination shell, we advance the hypothesis that the backbone of Dy’s ligands acts indirectly through a polarization of the first coordination shell’s local charge. In other words, the acac π system can allow a strong polarization of the CO bonds. This idea is supported by fact that **vib2**, being characterized by a rigid motion of the ligands’s intramolecular bonds, do not modulate significantly the local charge distribution of the first coordination shell, while the carbonyl stretching motions characterizing **vib1** lead to local charges fluctuations two orders of magnitude larger (see Figure 6C).

The smoking gun of the role of charge polarization is provided by repeating the calculation of the spin-phonon coupling for **vib1** and **vib2** by fixing the computed LOPROP multipoles expansions to those of the equilibrium geometry. In the case of **vib2** no difference is observed with respect to the calculation done adapting the charges during the vibrational motion, while large deviations are observed for **vib1**. Interestingly, by removing the modulation of the atomic charges in **vib1**, the results become closer to those obtained by removing the acac ligand altogether (see Figure S7). Additional evidence of the correlation between electrostatic polarization and spin-phonon coupling is provided by the computation of the effect of atomic displacements on the molecular dipole moment. The results are plotted in Figure 6D, in the same way as for the atomically resolved spin-phonon coupling. With exclusion of the Dy atom, the two plots correlate very nicely, further supporting the electrostatic mechanism for nonlocal spin-phonon coupling.

It is important to remark that although all these considerations do not take into account the thermal population of vibrations and the resonance conditions imposed by eqs 3 and 4, the discussion of **vib1** and **vib2** is crucial to highlight the origin of spin-phonon coupling in lanthanide ions beyond the simple argument of the relevance of distortions affecting the first coordination shell. Moreover, the spin-phonon coupling intensity of high-energy vibrations as the ones discussed here, would determine the τ_0_ of the Orbach process of molecules showing high axiality and large splitting of the electronic levels. Although this is not relevant for explaining the relaxation behavior of **Dyacac** (*vide infra*), it becomes of crucial importance for the design of new high-performance lanthanide SMMs with values of *U*_eff_ above 1000 cm^–1^.

Finally, we turn to the discussion of the modes directly involved in the spin–lattice relaxation of **Dyacac** due to the constraints introduced by thermal population and resonance conditions. The latter is evidenced in the right panel of Figure 5, where the energy of the computed spin levels are superimposed to the spin-phonon coupling diagram of the low energy vibrations. The modes close in energy to the second excited KDs, responsible for high-temperature relaxation, are rather delocalized on the molecular unit, but it is still possible to discern a significant contribution of ligand’s methyl groups rotations and torsions of the entire acac’s *sp*^2^ structure. These motions also accompany a modulation of the Dy–O bonds’ length and OO angles. Raman relaxation receives contributions from a large number of vibrations, however, at low temperature, only the first few modes are significantly populated and able to contribute. As discussed before, these modes are delocalized on the entire unit cell and are characterized by almost-rigid translations of the molecules in space overlapped to slight rigid rotations and intramolecular distortions. We note that the acacs’ Me group rotation are often involved in these mixed modes. This has been also observed for molecular qubits presenting acac ligands^37,41^ suggesting a critical role of this low-energy motion in promoting admixture of intra- and intermolecular motions.

## Discussion

The advent of SMMs with very large zero-field-splitting has led to a strong deviation of spin relaxation from the expected Orbach trend at low temperature. Since then, a Raman relaxation mechanism has been postulated to take place in that regime and to follow a phenomenological power-law τ^–1^ ∝ *T*^*n*^, with 2 < *n* < 6, regardless the standard picture of Raman relaxation theory predicts *n* = 9 for Kramers systems.^75^ Such a phenomenological approach, however, hides the underlying physical process responsible for relaxation and does not provide a clear indication on how we can to tackle the problem of fast Raman relaxation at low temperature. It has now been clearly demonstrated that the low energy vibrational DOS of molecular crystals significantly deviates from the Debye model and that optical modes appear at surprisingly low energies. Under these circumstances, the temperature dependence of Raman relaxation across degenerate KDs is shown to follow the Fourier transform of the two-phonon correlation function of pairs of degenerate phonons that are simultaneously absorbed and emitted from the low-energy vibrational spectrum, each contributing to τ^–1^ with a factor of
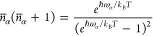
6In the limit of *k*_B_*T* < *ℏω*_α_, eq 6 reduces to the exponential expression τ ∼ exp(*ℏω*_α_/*k*_B_*T*), where *ℏω*_α_ is of the order of the first Γ-point optical modes. Summing up contributions from different phonons at different temperatures, eq 6 leads to the τ^–1^ ∝ *T*^*n*^, with 2 < *n* < 9.

Interestingly, in the limit of *k*_B_*T* > *ℏω*_α_, eq 6 leads to τ ∼ *T*^–2^, which has been recently shown to dominate high-temperature relaxation in *S* = 1/2 molecular qubits.^36^ It must be noted, however, that even though the phonon contribution to Raman relaxation in *S* = 1/2 and high-spin SMMs follows the same *T*-dependence, the origin of spin-phonon coupling is qualitatively different. For *S* = 1/2 systems in external field or in the presence of dipolar/hyperfine fields, Kramers theorem does not prevent the transition between the *m*_*S*_ = ± 1/2 states, and Raman relaxation is due to the quadratic dependence of the spin Hamiltonian with respect to atomic displacements.^36,76^ Instead, zero-field Raman relaxation in high-spin Kramers SMMs depends on the linear term in the spin-phonon coupling with respect to the phonon variables, but at the second order in time-dependent perturbation theory, which is made possible by low-lying excited KDs states, as depicted by eq 4. The identification of the correct relaxation mechanism is not only crucial for the correct interpretation of relaxation times but also provides a clear pathway to understanding how spin-phonon coupling arises from molecular motions and electronic structure. Indeed, in this contribution it has been possible to analyze the spin-phonon coupling on a single-vibration basis and reveal that both modulations of the geometry of the first coordination sphere, as well as electrostatic polarization effects, are at the origin of the coupling of the spin with the lattice.

On the basis of these new findings, it is finally possible to draw an updated list of requirements for disengaging the magnetic molecular degrees of freedom from the vibrational thermal bath. Many of the guidelines that will follow have already been suggested by others in the context of slowing down the Orbach mechanism or the magnetization tunneling, and here we will provide a comprehensive view of their effect on Raman relaxation. There are three different aspects that pertain to spin-phonon relaxation: (1) the nature and symmetry of the static crystal field, (2) the population of phonons participating to relaxation, and (3) their spin-phonon coupling value. Each of these three aspects can be acted upon by implementing the following guidelines:

1a. Kramers ions with high *J* values^10,22^
1b. Large crystal field splitting of the ground *J* multiplet^77,78^
1c. Crystal field operator of eq 2 with vanishing transverse (*m* ≠ 0) terms, not limited to the *g*-tensor of the ground state KD^77,79,80^
2a. Low vibrational density of states at the spin resonance energies^40^
2b. Small amount of low-energy vibrations in both the lattice and the molecular unit^31,34,45^
3a. Rigid ligands able to decouple intramolecular motions from low-energy acoustic vibrations^31,35^
3b. Use of ligands with donor atoms’ local charge not affected by local vibrations

Although all these rules apply to both Orbach and Raman relaxation, the efficiency of their implementation will generally be different for the two mechanisms. For instance, enforcing the points 1b and 1c will increase the value of the KD doublets mediating relaxation. The latter quantity is directly linked to the energy of the phonon mediating Orbach relaxation, thus influencing relaxation rate exponentially, but it only enters to the power of two in the definition of the Raman rate prefactor (see eqs 3 and 4). As a consequence, if increasing the relaxation time at low temperature is the goal, points 1b and 1c are probably not sufficient because the Raman process will still dominate the relaxation. This is also a consequence of the fact that the energy of the phonons contributing to Raman relaxation is not necessarily in resonance with the spin states and low-energy vibrations will always dominates the two-phonon relaxation. Raman relaxation, however, will be more sensitive to the guidelines 3a and 3b. These measures act to reduce the effective coupling of spin to low-energy vibrations, which enters to the fourth power into the relaxation rate expression instead of to the second in the case of Orbach.

This series of new guidelines to control spin relaxation has been obtained on the basis of a small number of studies and much more work will be necessary in order to completely explore the relation between spin-phonon relaxation and the chemical structure of coordination compounds. Although we can confidently claim that the physical principles behind spin relaxation have now been elucidated, we must stress out that correctly framing these guidelines into a chemical language is the next big challenge toward engineering new SMMs. In this regard, it is important to note that *ab initio* simulations have played a key role in the process of optimizing molecular’s zero-field splitting (rules 1a–c), and we believe that the same also have the potential to guide synthetic efforts in the implementation of rules 2a–3b. For instance, we propose that rules 2a–3a could be optimized by means of a strategy based on increasing the value of the frequency of the first optical phonon mode of the crystal’s unit cell, which can be easily obtained from periodic-DFT calculations at the Γ-point level of approximation.

Regarding possible implementation of rule 3b, we can state that the electrostatic polarization of the donor atom with respect to the vibrational modes strongly affects the coupling with the spin. Moreover, we expect that such a phenomenon is enhanced for ligands where many resonance contributors involving the donor atoms can be drawn. Indeed, conjugated *sp*^2^ bonds also allow atoms beyond the second-coordination sphere to efficiently modulate the donor’s charge in virtue of electronic delocalization and, therefore, contribute to spin-phonon coupling. These compounds are more prone to have significant spin-phonon couplings of vibrations connecting the first and *n*th coordination sphere (see the Supporting Information for a comparison among acac, acetone, and 2-propanol, Figure S13). Such a guideline provides further emphasis to the importance of exploring organometallic compounds based on haptic ligands, which might combine high rigidity (2b,3a) and low level of atomic polarizability (3b) induced by vibrational activity. In this last case, the resonance formulas that can be drawn are equivalent from the point of view of charge delocalization on the donors and, as a consequence, the relaxation induced by charge modulation should not play a major role. We expect such a strategy to be already accounted for in the class of dysprosocenium SMMs^26,28,81^ and similarly designed molecular spin qubits.^82,83^ Finally, we remark that these considerations are likely to affect more strongly the high-temperature Orbach regime of SMMs with large *U*_eff_, where relaxation proceed through the absorption of phonons with energy comparable to the stretching of rigid bonds such as those active in **vib1**.

In conclusion, we have applied *ab initio* spin dynamics to the case of a Dy-SMM and successfully explained the origin of its slow spin relaxation. Our method provided the first successful prediction of both one- and two-phonon relaxation rates in a lanthanide compound free from any adjustable parameter and allowed to completely bypass phenomenological approaches commonly used in the field. The identification of the correct mechanism of relaxation made it possible to derive an updated list of requirements to slow down both Orbach and Raman relaxation. In particular, from the analysis of *ab initio* vibrations and spin-phonon coupling coefficients, it was possible to derive new insights on the molecular quantities that regulate relaxation rates across all temperature ranges. Of particular relevance is the finding that electrostatic polarization plays an active role in determining spin-phonon coupling, offering a new chemically sound pathway to control spin relaxation.

## Supporting Information Available

Supporting data and videos (ZIP)
Experimental and computational methods, details on modeling of electrostatic interactions, convergence tests for the computed relaxation time, details on the sample synthesis and characterization with crystallography and magnetometry (PDF)

## Notes

All relevant data discussed in this paper are available from the authors upon request.
