# Supplementary material for: A Complete Ab Initio View of Orbach and Raman Spin–Lattice Relaxation in a Dysprosium Coordination Compound
Source: J Am Chem Soc. 2021 Aug 16;143(34):13633–45. doi: 10.1021/jacs.1c05068 (PMC8414553; doi:10.1021/jacs.1c05068)
Supplement: Supplementary file 2 — ja1c05068_si_002.pdf [file ja1c05068_si_002.pdf]

Supporting Informations for: A complete ab initio view of Orbach and Raman spin-lattice relaxation in a Dysprosium coordination compound

Matteo Briganti,[†] Fabio Santanni,[†] Lorenzo Tesi,[†] Federico Totti,[†] Roberta Sessoli,[*,†] and Alessandro Lunghi[*,‡]

†*Department of Chemistry "Ugo Schiff", INSTM Research Unit, Università degli Studi di Firenze, Sesto F.no, Italy*

‡*School of Physics, AMBER and CRANN Institute, Trinity College, Dublin 2, Ireland*

E-mail: roberta.sessoli@unifi.it; lunghia@tcd.ie

Contents

S1 Computational and Experimental Methods **S3**

S1.1 Unit-Cell Optimization and Vibrational Spectrum Calculation S3

S1.2 Multi-reference Electronic Structure Calculations S3

S1.3 Calculation of the Spin-Phonon Coupling Coefficients S4

S1.4 Spin-Phonon Relaxation Simulations S5

S1.5 Synthesis and Characterization S6

S1.6 Cantilever Torque Magnetometry S6

S1.7 Magnetization Dynamics S7

S2 Computational Details **S9**

S2.1 Electronic structure modelling. S9

S2.2 Computed Normal Modes S18

S2.3 Electrostatic modelling of spin-phonon coupling. S20

S2.4 Derivatives of Electric Dipoles S23

S2.5 Convergence of relaxation time. S25

S2.6 Effect of anharmonic phonons. S27

S2.7 DFT Calculations on Isolated Ligands S30

S3 Experimental Details **S31**

References **S48**

S1 Computational and Experimental Methods

S1.1 Unit-Cell Optimization and Vibrational Spectrum Calculation

Crystal geometry optimization and simulation of Γ-point vibrations were carried out with the software CP2K.[1] Gaussian plane waves formalism (GPW)[2] was employed to solve the Kohn-Sham problem. Norm-conserving Goedecker-Tetter Hutter (GTH) pseudopotentials,[3–5] along with double zeta basis set with polarization functions (DZVP-MOLOPT-SR) were employed for all atoms. The GGA functional PBE[6] with D3(BJ) empirical dispersion corrections[7–9] was employed. A wave-plane cut-off of 400 Ry was applied. Cell optimization was performed employing a very tight SCF convergence criteria of $1 \cdot 10^{-9}$ for the wave-function gradient. Similarly, the threshold on the maximum force gradient for the cell and geometry optimization was set very to the very high standard of $1 \cdot 10^{-7}$ Hartree/Bohr. The experimental and optimized cell parameters are listed in table S1 and show a deviation as little as 1.5% for the lattice vector $|a|$, and lower than 1% for the other lattice parameters. Once the cell had been optimized, the phonon spectrum at the Γ-point was computed by estimation of the Hessian matrix with a finite differences method, employing a step of ±0.01 Å.[10]

S1.2 Multi-reference Electronic Structure Calculations

Complete Active Space Self Consistent Field (CASSCF) calculations[11,12] followed by Complete Active Space State Interaction (CASSI-SO) to introduce spin-orbit effect,[13] were performed in order to compute the electronic structure of our proposed models. All calculations were performed with MOLCAS 8.0 quantum chemistry software.[14,15] SINGLE_ANISO subroutine was employed to extract ESO parameters from the multiconfigurational calculation.[16,17] The active space consisted in the 7 4f orbitals with 9 electrons, CAS (9,7). Calculations were performed averaging on all the 21 sextets of the ground ^6H multiplet. Electric field multipole expansions were extracted within the LOPROP approach[18] on the final Spin-

Orbital wavefunction. The ANO-RCC basis sets employed[19,20] are shown in Table S2.

From the optimized coordinates of the unit cell by pDFT, the molecular cluster to be employed for the spin phonon coupling calculation was extracted. Our proposed approach was the same one that some of us applied to the DyDOTA complex in a previous publication:[21] the combination of i) a molecular cluster which is explicitly treated from the quantum chemical point of view, *i.e.* each atom represented by a set of gaussian-type atomic orbitals, and ii) a large number of point charges computed at the periodic level embedding the explicit molecular cluster in order to simulate the periodic Madelung electrostatic potential inside the crystal. Density Derived Atomic Point Charges (DDAPC)[22] were extracted from the final geometry to mimic the Madelung potential. Tests reported in SI show that a 7x5x7 supercell of point charges gives well converged electronic structure results.

S1.3 Calculation of the Spin-Phonon Coupling Coefficients

The spin-phonon coupling coefficients were computed within the methodology published on previous works.[23,24] In a nutshell, the molecular geometry is distorted along every Cartesian degree of freedom, X_{is}, where the index i runs on the atoms of the molecule and s runs on the three Cartesian directions. We apply distortions of between -0.1 Å and 0.1 Å with step 0.02 Å for a total of ten points per degree of freedom. We determine the Crystal Field coefficients B^l_m for each of them, for a total of 1830 CASSCF calculations. Cartesian spin-phonon coupling coefficients, namely $(\partial B^l_m/\partial X_{is})$, are computed as the linear term of a third-order interpolation of the profile B^l_m vs X_{is}. The atomically-resolved spin-phonon coupling coefficients of Fig. 6C are computed as $\sum_s \sum_m (\partial B^l_m/\partial X_{is})^2$, similarly to the atomically-resolved dipole moment derivatives, which are computed as $\sum_s \sum_t (\partial \mu_t/\partial X_{is})^2$, where t runs over the three components of the dipole moment, $\vec{\mu}$. Mode-resolved spin-phonon coupling coefficients, $(\partial B^l_m/\partial q_\alpha)$, are computed as

$$\left(\frac{\partial B^l_m}{\partial q_\alpha}\right) = \sum_{is} \sqrt{\frac{\hbar}{2\omega_\alpha m_i}} L_{[3(i-1)+s],\alpha} \left(\frac{\partial B^l_m}{\partial X_{is}}\right) , \tag{S1}$$

where L_{ij} are the elements of the Hessian's eigenvector matrix, and m_i is the mass of atom i. Finally, the spin-phonon coupling distribution is computed as $\left[\sum_m \left(\partial B^l_m/\partial q_\alpha\right)^2\right](\omega_\alpha)$.

S1.4 Spin-Phonon Relaxation Simulations

One-phonon relaxation rate was computed employing the full Redfield's Liuvillian[10]

$$
\begin{aligned}
R_{ab,cd} = -\frac{\pi}{\hbar^2}\sum_\alpha \Big\{ \sum_j & \delta_{bd} V^\alpha_{aj} V^\alpha_{jc} G^{1-ph}(\omega_{jc}, \omega_\alpha) \\
& - V^\alpha_{ac} V^\alpha_{db} G^{1-ph}(\omega_{db}, \omega_\alpha) \\
& - V^\alpha_{ac} V^\alpha_{db} G^{1-ph}(\omega_{ca}, \omega_\alpha) \\
& + \sum_j \delta_{ca} V^\alpha_{dj} V^\alpha_{jb} G^{1-ph}(\omega_{jd}, \omega_\alpha) \Big\} \ ,
\end{aligned} \tag{S2}
$$

where only secular terms are retained, namely the elements of $R_{ab,cd}$ that fulfill $\omega_{ac}+\omega_{db} = 0$. The terms $R_{aa,bb}$ correspond to the W^{1-ph}_{ab} rates of Eq. 3 and V^α_{ab} is a shorter notation for $\langle a|\hat{V}_\alpha|b\rangle$.

Two-phonon relaxation rates were computed using Eq. 4, together with the full expression of $G^{2-ph}_\pm$, which reads

$$
\begin{aligned}
G^{2-\mathrm{ph}}_{+} = & \delta(\omega - \omega_\alpha + \omega_\beta)\bar{n}_\alpha(\bar{n}_\beta + 1) + \\
& \delta(\omega + \omega_{\alpha\mathbf{q}} + \omega_\beta)(\bar{n}_\alpha + 1)(\bar{n}_\beta + 1) \ ,
\end{aligned} \tag{S3}
$$

$$
\begin{aligned}
G^{2-\mathrm{ph}}_{-} = & \delta(\omega + \omega_\alpha - \omega_\beta)(\bar{n}_\alpha + 1)\bar{n}_\beta + \\
& \delta(\omega - \omega_\alpha - \omega_\beta)\bar{n}_\alpha\bar{n}_\beta \ .
\end{aligned} \tag{S4}
$$

Differently from Eq. S2, W^{2-ph} does not account for population-coherence transfer and coherence-coherence transfer processes, namely $\rho_{aa} \to \rho_{ab}$ and $\rho_{ab} \to \rho_{cd}$, where $\hat{\rho}$ is the reduced spin density matrix. We note that the exclusion of these terms is an approximation

to the formalism of Raman relaxation, which effect on predicted times should be further explored.

$R_{ab,cd}$ and W_{ab}^{2-ph} are $(2J+1)^2 \times (2J+1)^2$ and $(2J+1) \times (2J+1)$ matrices, respectively. After diagonalization, they are always found to have a zero eigenvalue corresponding to the equilibrium state. The smallest, non-zero eigenvalue, instead, corresponds to a population transfer within the ground state KD and it is interpreted as the relaxation rate τ^{-1}. Dirac deltas appearing in the relaxation rate's expressions are approximated with a Gaussian function with a $\sigma = 10$ cm^{-1}. We note that relaxation time in **Dyacac** is dominated by the spin-phonon coupling coefficients with $l = 2$.

S1.5 Synthesis and Characterization

Compounds $[Dy(acac)_3(H_2O)_2]\cdot EtOH\cdot H_2O$ (**Dyacac**) (acac = acetylacetonate), and its diamagnetic diluted analogous $[Y_{0.9}Dy_{0.1}(acac)_3(H_2O)_2]\cdot EtOH\cdot H_2O$ (**Dy$_{0.1}$acac**) were obtained by employing the synthetic strategy reported in literature[25] (see SI for further details). According to literature, the products are isostructural and crystallize in the monoclinic P21/n space group, in which the asymmetric unit is composed by a $[Dy(acac)_3(H_2O)_2]$ molecule and by ethanol and water crystallization molecules (green unit in Fig. S14). The crystallographic unit thus contains two magnetically non-equivalent molecules (related by a glideplane or a binary screw axis) and their centrosymmetric analogues (Fig. S14). The Dysprosium ion is coordinated by eight oxygen atoms, six of them belonging to the acetylacetonate ligands, and the remaining two from coordinated water molecules. The coordination geometry might be associated to a pseudo-dodecahedron, with an approximative D_{2d} local symmetry (Fig. S15), as computed by SHAPE 2.1[26] with a CShM value of 0.167.

S1.6 Cantilever Torque Magnetometry

The two rotation measurement sets, Rot1 and Rot2, were performed at various temperature values, from 10 to 200 K, and magnetic fields, from 0.5 T, at low temperature, to 7.5 T at

high temperature (see Fig. 2). All results are displayed in Fig. S16-S20). The rotation axis for Rot1 has an estimated angular deviation of 25° from the crystallographic b axis. As a result, the plane ac* can be considered mostly probed during rotation. At 10 K, the torque momentum is zero at 87° and 170°, while at 200 K there is a shift to lower values, *i.e.* 70° and 154°. This shift in temperature is attributed to a change of the magnetic anisotropy of the excited spin states, with respect to the ground state. A second aspect to mention is the angle separation between the zeroes, which is 83° for 10 K and 84° for 200 K. At first glance, this is in contrast with the relation $t_Y \propto \sin(\eta)\cos(\eta)$, where η is the angle between the projection of the principal axes of the molecular anisotropy and B_Z, which imposes a null torque momentum with 90° periodicity. However, two magnetically non-equivalent molecules are present in the crystal cell. The measured t_Y is therefore the result of the sum of the contributions coming from the two molecules. In Rot2, the rotation axis coincides with c*, for which the *ab* plane is investigated. The zeroes are found at 25° (along b) and 115° (along a), independently on temperature and fulfilling the 90° separation. This agrees with the fact that in a monoclinic system the crystallographic b axis must coincides with one of the principal magnetic anisotropy axes and that the two sets of magnetically non-equivalent have identical contribution.

S1.7 Magnetization Dynamics

Magnetization dynamics of a microcrystalline powder sample of **Dy$_{0.1}$acac** was probed by Alternate Current (AC) susceptometry. The sample was prepared and introduced into the instrument in such a way that no desolvatation occurred (see Experimental Details). Measurements of the real (χ') and imaginary (χ") components of the susceptibility were performed by sweeping the frequency of the AC magnetic field between 1 Hz and 10 kHz for various temperatures (6-15.5 K) for two external magnetic field values (0 and 50 mT). AC susceptometry measurements below 6 K were not possible because of the limited frequency window of the instrument. On the other hand, the relaxation time in this temperature range

is too short for measurements of the magnetization decay. Measurements up to room temperature were not performed to avoid degradation of the sample, however by plotting the real component of the susceptibility extracted from the measurements at 0 mT as $\chi' T$ versus T, we observe the limit value of 13.8 $cm^3\ mol^{-1}$ K for the lower frequencies (Fig. S21). This value well agrees with the limit value of 14.17 $cm^3\ mol^{-1}$ K expected for Dy^{3+} according to the Curie law. Relaxation time constants (τ) were extracted by fitting χ" as a function of AC frequency with a modified Debye model extended to include two relaxation time contributions (Eq. S10, Fig. S22).[27,28] The need for two contributions is to be attributed to the nature of the sample, resulting from the non-standard preparation procedure employed. The main contribution is the faster one, and its temperature dependence overlap with the relaxation times reported in literature for the same compound.[25] The experiment was repeated by applying 50 mT of external magnetic field, but no differences are present with respect to the zero field measurements, suggesting that pure tunneling is not relevant in the investigated temperature range (Fig. S23). In first approximation, the temperature dependence of τ, reported as $ln(\tau)$ vs. T^{-1} in Fig. S25, can be reproduced considering an Orbach relaxation process occurring at higher temperature plus a second empirical term which accounts for the Raman relaxation mechanism.[29] Using this rough model, we can extract an energy barrier U of 139 ± 8 cm^{-1}. The set of parameters obtained from the fit is reported in Fig. S25.

Hysteresis measurements were also performed on **$Dy_{0.1}$acac**. A butterfly-shaped hysteresis loop was recorded at 2 K with a magnetic field speed of 250 mT/min. (Fig. S26). The closure of the hysteresis at zero field is due to QTM, relevant at this temperature. As the field increases the hysteresis opens, as the QTM is more and more suppressed (see Experimental Details). An estimation of the long relaxation time at 2 K, as a function of the magnetic field (Fig. S27), can be obtained from the hysteresis data. By applying eq. S11 (see section S3), it is possible to estimate a lower limit value of $\tau = 0.8$ s at zero field, where the QTM is still contributing to the magnetization dynamics, and an upper limit value of 11.5 s at $B = 1500$ Oe. Indeed, the highest value of τ is in reasonably agreement with the *ab*

initio computed value, suggests an almost complete suppression of QTM, whose contribution is not accounted for in the theoretical model.

S2 Computational Details

S2.1 Electronic structure modelling.

The asymmetric unit of **Dyacac** is composed by one $Dy(acac)_3(H_2O)_2$ complex and two co-crystallized solvent units: one ethanol and one water molecules, while the crystal unit cell presents four crystallography equivalent $Dy(acac)_3(H_2O)_2$ molecules, along with other 8 co-crystallized solvent molecules. From the optimized coordinates of the unit cell by pDFT, the molecular cluster to be employed for the spin phonon coupling calculation was extracted.

Table S1: Experimental and optimized cell parameters by CP2K of **Dyacac**

	a	b	c	α	β	γ
**Exp**	10.6461(2)	19.8673(4)	11.0707(2)	90.00	92.2270(9)	90.00
**Opt**	10.405	19.575	10.909	89.931	91.939	89.603

Table S2: Employed ANO-RCC basis sets[19,20] and contractions along all the CASSCF/CASSI-SO calculations

ATOM	LABEL	BASIS SET	CONTRACTION
DY	VTZP	[25s22p15d11f4g2h]	[8s7p5d3f]
O	VTZP	[14s9p4d3f2g]	[4s3p2d1f]
C	VDZP	[14s9p4d3f2g]	[3s2p1d]
H	VDZ	[8s4p3d1f]	[2s]

The crystal structure optimization has been performed without symmetry constraints. As a consequence, the four structurally equivalent molecules inside the crystal cell show slightly different structural parameters after the optimization procedure. However, the Dy-O bond lengths in the first coordination sphere show a variation below 3% (see table S3), and CASSCF/CASSI-SO calculations performed on two of the four molecules show an average error around 2% on the electronic structure parameters (ground state g-tensor and energy

ladder, see table S4). As a consequence, for the simulation of the spin-lattice relaxation the four molecules have been considered equivalent as they are in the experimental the crystal structure, and only one molecule has been extracted for the following calculation of the spin-coupling parameters.

Table S3: Dy-O bond lengths in Angstroms of the first coordination shell for the four molecules in the crystal cell in the optimized crystal structure

Dy-OX	Mol1	Mol2	Mol3	Mol4
O1	2.314	2.359	2.320	2.347
O2	2.432	2.440	2.436	2.431
O3	2.336	2.347	2.362	2.326
O4	2.375	2.365	2.374	2.408
O5	2.372	2.369	2.379	2.354
O6	2.400	2.383	2.401	2.417
O7	2.405	2.347	2.370	2.371
O8	2.392	2.318	2.318	2.320

Table S4: Energy ladder and ground state g-tensor computed at the CASSCF/CASSI-SO level of theory for the two optimized structure of the two magnetically inequivalent molecules in the crystal cell.

	Mol1	Mol2	error (%)
	Ground State g-tensor		
g_X	0.0299	0.0295	1.3
g_Y	0.0529	0.0497	6.2
g_Z	19.4418	19.3464	0.5
	Energy ladder (cm^{-1})		
E_0	0.000	0.000	-
E_1	117.751	117.114	0.5
E_2	158.647	160.746	1.3
E_3	197.964	193.938	2.1
E_4	250.738	242.446	3.4
E_5	339.033	360.736	6.2
E_6	413.239	417.029	0.9
E_7	517.611	511.103	1.3

Our proposed approach was the same one that some of us applied to the $[Dy(DOTA)H_2O]^-$ complex in a previous publication: the combination of i) a molecular cluster which is explicitly treated at the quantum chemical point of view, *i.e.* each atom represented by a

set of gaussian-type atomic orbitals, and ii) a large number of point charges computed at the periodic level embedding the explicit molecular cluster in order to simulate the periodic Madelung electrostatic potential inside the crystal. In order to choose the most reliable computational model, two main issues need to be faced. Each issue is strictly related to the two components of the model: i) the best choice of the molecular unit to be treated explicitly in the post-HF calculation to compute the spin-orbit coupling coefficients, ii) the best representation of the Madelung potential in the hydrogen bond framework of the molecular crystal.

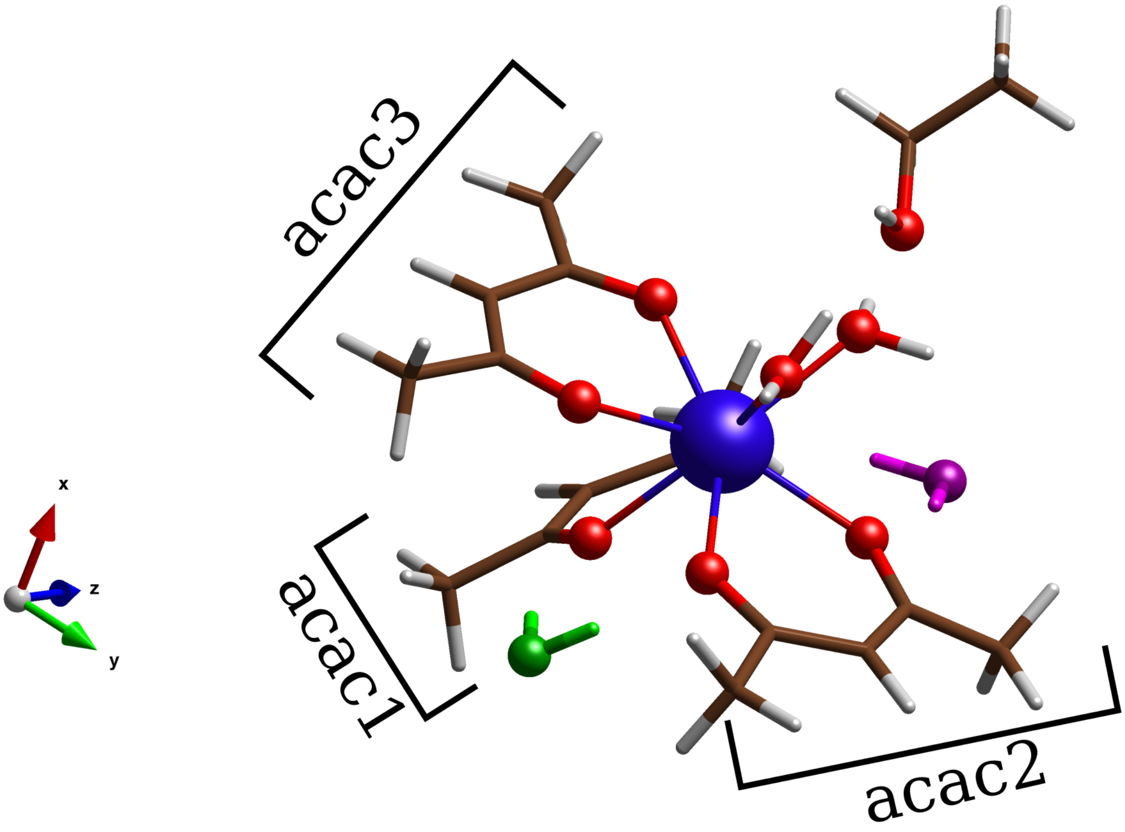

Fig. S1: **Dyacac** models: Dy, O, C, H are blue, red , brown, white, respectively. The green water molecule is the co-crystallized solvent water molecule which is present in models **M1** and **M3**, while the purple one is the one belonging to a neighbour $Dy(acac)_3(H_2O)_2$ complex in the crystal, which is present in models **M2** and **M3**

In order to address the first issue, three molecular clusters have been attempted. Each

model includes the $Dy(acac)_3(H_2O)_2$ complex and the ethanol solvent co-crystallized molecule. The models differed only by the further explicitly included water molecules (green and purple in Figure S1). Indeed two water molecules, even if not directly coordinate to the Dy atom, are very close to it inside the crystal packing: one belongs to a neighbor $Dy(acac)_3(H_2O)_2$ complex inside the crystal cell (purple molecule in Figure S1), while the other is the water solvent co-crystallized molecule (green molecule in Figure S1). Two models were therefore built with only one of the two molecules (**M1** and **M2**, see Figure S1) and the last one with both molecules (**M3**, see figure S1). The three models have been evaluated when embedded in 3x3x3 cell of point charges (vide infra). From the results (see Table S5), it appears that only one of the two water molecules is effectively necessary in order to reproduce the energy ladder of the 'complete' **M3**: the molecule which is present in **M1** (green one in Figure S1). As consequence, **M1** was chosen to perform the following spin-phonon coupling calculation.

Table S5: Energy ladder for the ground $^6H_{\frac{15}{2}}$ multiplet in cm^{-1}, ground Kramers'doublet value and its direction in the crystal frame of Dy^{3+} ion computed for the three models **M1-3** embedded in a 3x3x3 supercell of point charges

	**M1**	**M2**	**M3**
E_0	0.000	0.000	0.000
E_1	100.884	93.557	99.381
E_2	129.108	122.408	126.734
E_3	171.141	160.669	167.696
E_4	216.923	197.846	209.031
E_5	290.764	267.923	278.979
E_6	352.214	316.866	333.529
E_7	450.465	417.253	431.324
g_z	19.417	19.429	19.442
a	-0.407851	-0.414557	-0.411872
b'	0.875374	0.889250	0.885058
c*	0.259574	0.193332	0.216875

The second issue has been faced by embedding our molecular complex in a supercell of atomic point charges computed at the pDFT level. Several dimensions of the supercell were evaluated (see table S6). Observing the energy ladder, beyond the 7x5x7 supercell (59719 point charges!) the energy spacing between levels suffers only minor changes (less than 1

cm^{-1}) and, as a consequence, it was choosen as a god compromise between accuracy and speed of the calculation.

Table S6: Energy ladder for the ground $^6H_{\frac{15}{2}}$ multiplet in cm^{-1}of Dy^{III} ion, model **M1**, computed for different dimensions of point charges supercell.

**SuperCell**	3x3x3	5x3x5	7x3x7	7x5x7	9x5x9	11x5x11	11x7x11	13x7x13	15x7x15
**N. of Point Charges**	6527	18239	35807	59719	98759	147559	206607	288591	384239
E_0	0	0	0	0	0	0	0	0	0
E_1	100.884	101.127	101.448	102.52	102.439	102.406	102.854	102.771	102.688
E_2	129.108	129.118	129.296	130.331	130.2	130.125	130.589	130.48	130.372
E_3	171.141	170.916	170.976	172.141	171.938	171.804	172.351	172.197	172.056
E_4	216.923	216.369	216.242	217.457	217.169	216.963	217.571	217.368	217.192
E_5	290.764	290.367	290.337	291.751	291.463	291.26	291.95	291.736	291.534
E_6	352.214	351.467	351.29	353.005	352.6	352.309	353.17	352.883	352.599
E_7	450.465	449.944	449.817	451.23	450.903	450.666	451.378	451.144	450.897

In summary, from our 'static' evaluation for the spin-phonon coupling calculation, **M1** was tested, and it was embedded in 7x5x7 supercell of point charges. Within this model, the effects on the crystal field parameters of the hydrogen displacement of the removed water molecule in model **M2** were evaluated in order to see if **M1** were a reliable choice also for the spin-phonon calculation. An almost negligible effect of that atoms' displacement was indeed observed on the energy splitting (see Figure S2). Such a test confirmed out previous assumption that **M1** was a good approximation even regarding the spin-phonon coupling.

Finally Dy displacements along the crystallographic a direction on **M1** embedded in two different supercells of point charges, the chosen one, 7x5x7, and the biggest one, 15x7x15, were evaluated in order to observe if different dimensions of the point charges supercell could affect the spin-phonon coupling. How it can be observed in Figure S3, the variation of the crystal field parameters as a function of the displacement is the same, and for each of the 27 parameters the curves are almost superimposable. Such tests confirmed the reliability and the goodness of our model also for the calculation of the derivatives of the crystal filed parameters as a function of the atomic displacements, i.e. to evaluate the spin-phonon coupling.

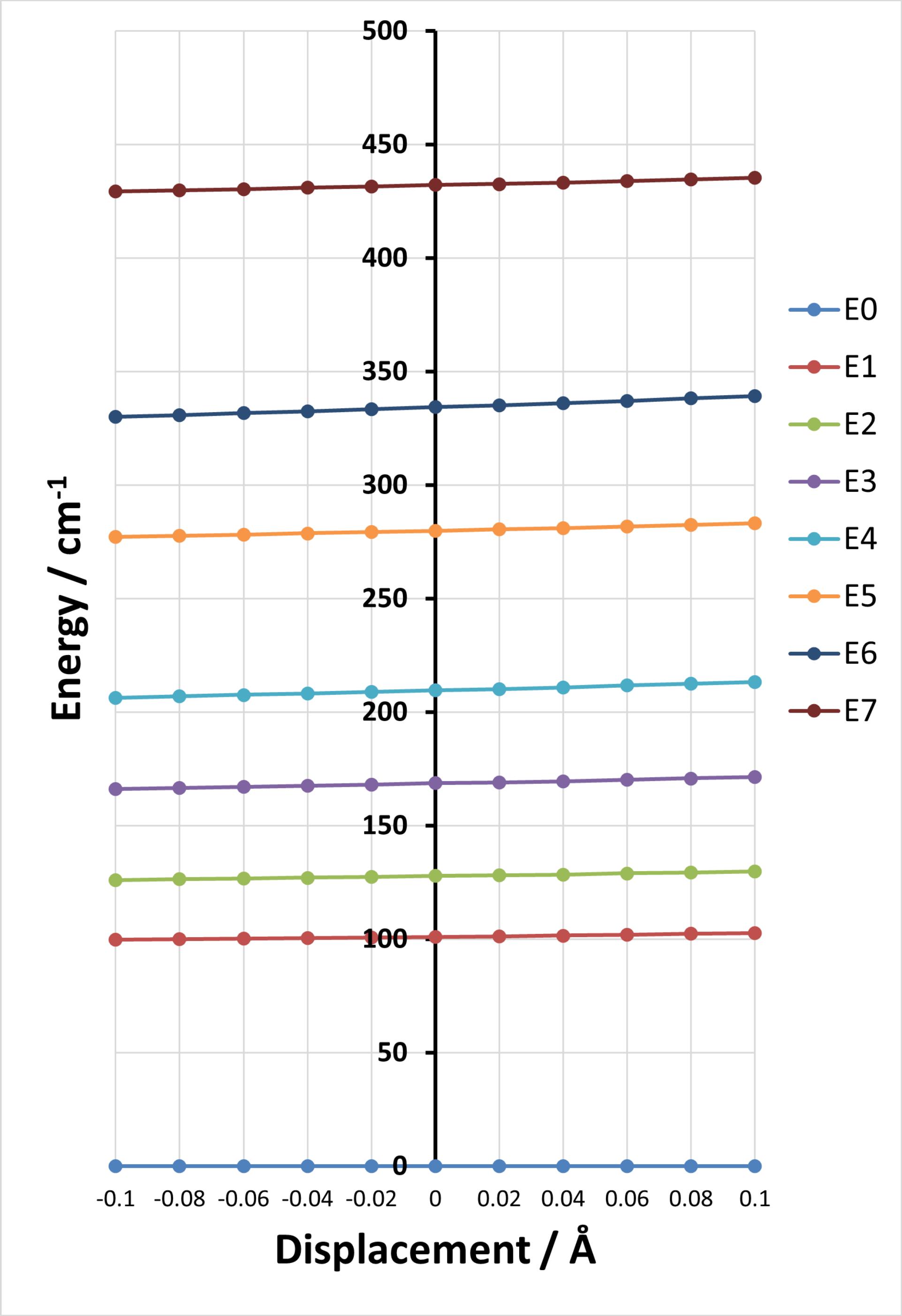

Fig. S2: Energy variation of the eight ground Kramers'doublets as a function of the Hydrogen displacement in **M2** embedded in a 7x5x7 cell of point charges

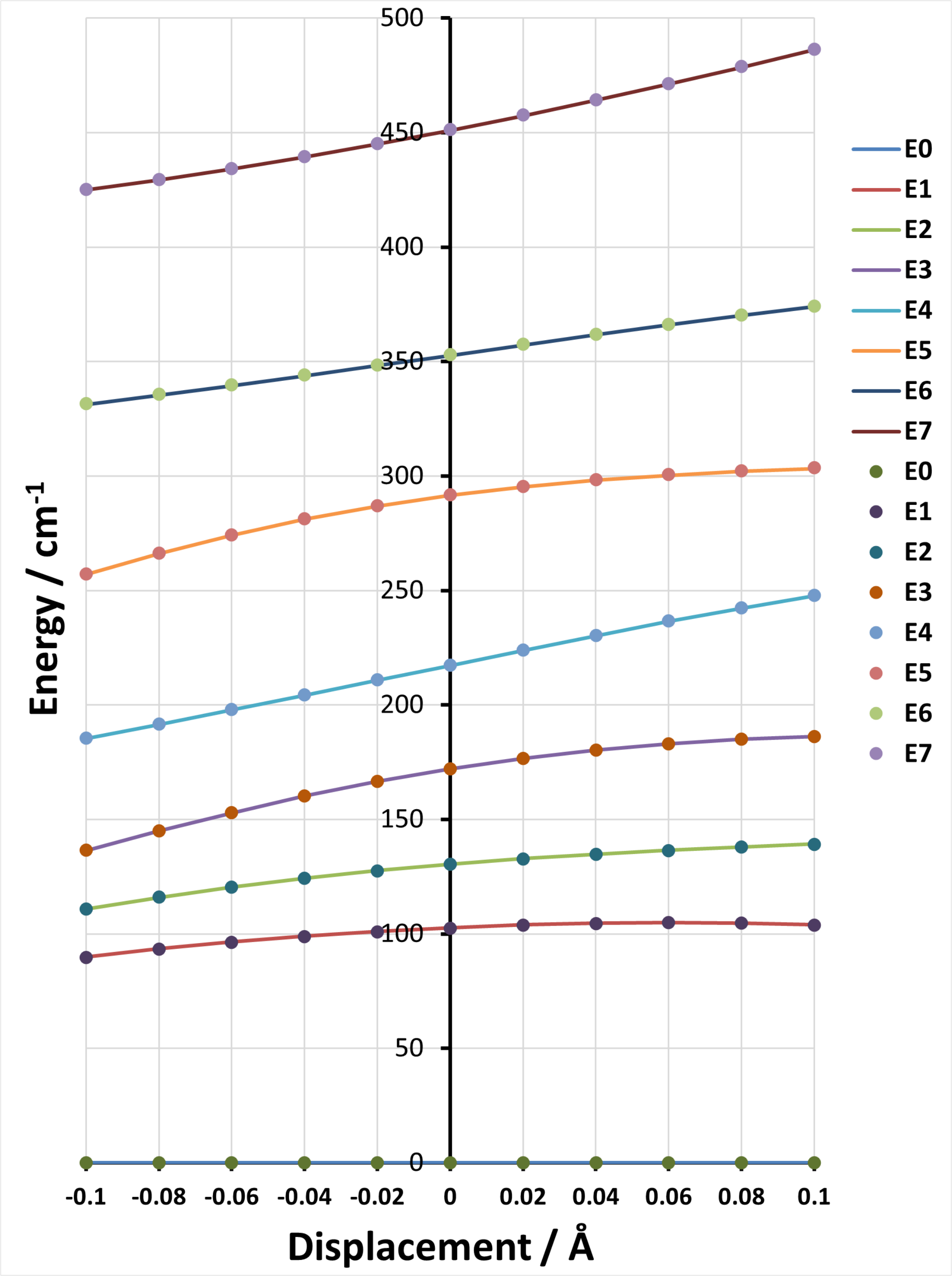

Fig. S3: Energy variation of the eight ground Kramers'doublets as a function of the Dy atom displacement along a direction in **M1** embedded in a 7x5x7 cell (points) and 15x7x15 (lines) cell of point charges.

Table S7: Extended Steves' Operators[30] in cm^{-1} computed within standard procedures[31–33] for the ground $^6H_{\frac{15}{2}}$ multiplet of Dy^{3+} ion, for model **M1**, computed in a 7x5x7 supercell of point charges.

l	m	O^l_m	l	m	O^l_m	l	m	O^l_m
2	-2	1.178598344504	10	-10	-0.000000014736	14	-14	-0.000000000004
2	-1	0.003109796981	10	-9	-0.000000003761	14	-13	0.000000000002
2	0	-0.985554058357	10	-8	0.000000013631	14	-12	0.000000000003
2	1	-2.171242701730	10	-7	0.000000018843	14	-11	-0.000000000001
2	2	3.396110119780	10	-6	-0.000000015620	14	-10	-0.000000000001
4	-4	-0.114545072420	10	-5	0.000000031050	14	-9	0.000000000001
4	-3	-0.048934590260	10	-4	0.000000013791	14	-8	0.000000000004
4	-2	-0.008366536368	10	-3	0.000000063300	14	-7	0.000000000003
4	-1	0.023549483722	10	-2	0.000000034223	14	-6	0.000000000004
4	0	0.030133335402	10	-1	-0.000000010518	14	-5	-0.000000000002
4	1	-0.017079355864	10	0	0.000000014322	14	-4	-0.000000000003
4	2	0.008109104825	10	1	-0.000000010970	14	-3	-0.000000000003
4	3	0.013890714112	10	2	-0.000000058405	14	-2	0.000000000000
4	4	0.041085352029	10	3	-0.000000043126	14	-1	0.000000000003
6	-6	-0.000584176315	10	4	-0.000000007915	14	0	-0.000000000002
6	-5	-0.001139345008	10	5	-0.000000007668	14	1	0.000000000001
6	-4	0.001315032430	10	6	0.000000004376	14	2	0.000000000003
6	-3	0.000518236870	10	7	0.000000024878	14	3	0.000000000004
6	-2	0.000320150159	10	8	0.000000013970	14	4	-0.000000000002
6	-1	-0.000083810297	10	9	-0.000000082642	14	5	-0.000000000003
6	0	-0.000384844594	10	10	-0.000000025062	14	6	0.000000000001
6	1	0.000542435938	12	-12	-0.000000000050	14	7	0.000000000001
6	2	-0.000510452704	12	-11	0.000000000006	14	8	0.000000000007
6	3	-0.000824141841	12	-10	0.000000000172	14	9	0.000000000001
6	4	0.000081427444	12	-9	-0.000000000152	14	10	-0.000000000001
6	5	-0.000327762090	12	-8	0.000000000147	14	11	0.000000000002
6	6	0.000011918616	12	-7	-0.000000000268	14	12	0.000000000001
8	-8	-0.000000744697	12	-6	-0.000000000107	14	13	-0.000000000001
8	-7	-0.000001260503	12	-5	-0.000000000035	14	14	-0.000000000001
8	-6	0.000000185007	12	-4	0.000000000230			
8	-5	-0.000001503128	12	-3	-0.000000000475			
8	-4	-0.000002262269	12	-2	-0.000000000612			
8	-3	-0.000002464488	12	-1	0.000000000240			
8	-2	-0.000001232844	12	0	-0.000000000140			
8	-1	0.000001039290	12	1	0.000000000287			
8	0	0.000000741544	12	2	0.000000000659			
8	1	-0.000002026124	12	3	0.000000000144			
8	2	0.000001194319	12	4	-0.000000000034			
8	3	0.000001544383	12	5	0.000000000154			
8	4	-0.000000230108	12	6	0.000000000111			
8	5	0.000000502549	12	7	-0.000000000254			
8	6	-0.000000701197	12	8	-0.000000000321			
8	7	-0.000000888416	12	9	0.000000000584			
8	8	-0.000000831361	12	10	-0.000000000154			
			12	11	-0.000000000353			
			12	12	-0.000000000097			

Table S8: Energy in cm^{-1} and g-tensors for the ground $^6H_{\frac{15}{2}}$ multiplet of Dy^{3+} ion, for model **M1**, computed in a 7x5x7 supercell of point charges.

Energy			a	b'	c*
0.000	g_X	0.044809858	-0.790771	-0.197256	-0.579458
	g_Y	0.088882564	-0.456903	-0.439741	0.773218
	g_Z	19.371140931	-0.407333	0.876195	0.257608
102.531	g_X	2.319774237	-0.780748	-0.033163	-0.623965
	g_Y	6.771127165	0.062608	-0.997717	-0.025312
	g_Z	12.445149231	-0.621701	-0.058828	0.781042
130.340	g_X	0.842921833	0.081911	0.945138	-0.316236
	g_Y	4.015014354	-0.879388	0.217858	0.423337
	g_Z	7.652474753	0.469007	0.243418	0.848988
172.148	g_X	1.682144786	0.358607	0.921101	-0.151571
	g_Y	1.708914734	-0.823588	0.388624	0.413128
	g_Z	11.374128016	0.439437	-0.023319	0.897971
217.460	g_X	0.133331054	-0.614883	0.628718	0.476059
	g_Y	0.496610784	-0.554039	-0.773984	0.306577
	g_Z	14.451752038	0.561212	-0.075246	0.824244
291.753	g_X	0.185458864	-0.238384	-0.172070	0.955806
	g_Y	0.345402331	0.715575	-0.696517	0.053077
	g_Z	15.651581936	0.656602	0.696603	0.289167
353.015	g_X	0.099673173	-0.539356	0.448863	0.712473
	g_Y	0.199974762	0.030261	-0.835211	0.549097
	g_Z	16.858639215	0.841534	0.317719	0.436893
451.243	g_X	0.018127091	0.159729	0.965795	-0.204273
	g_Y	0.032011129	-0.027736	0.211239	0.977041
	g_Z	19.209710972	0.986771	-0.150396	0.060529

S2.2 Computed Normal Modes

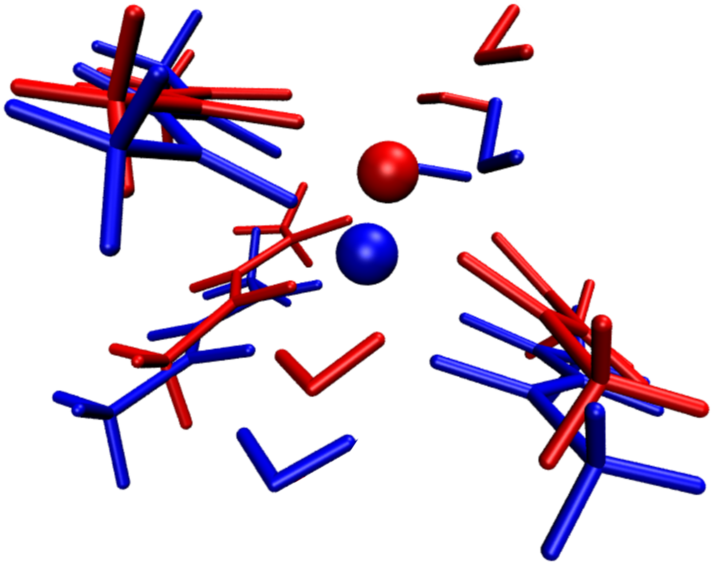

Fig. S4: Representation of the first vibration at 15.8 cm^{-1}.

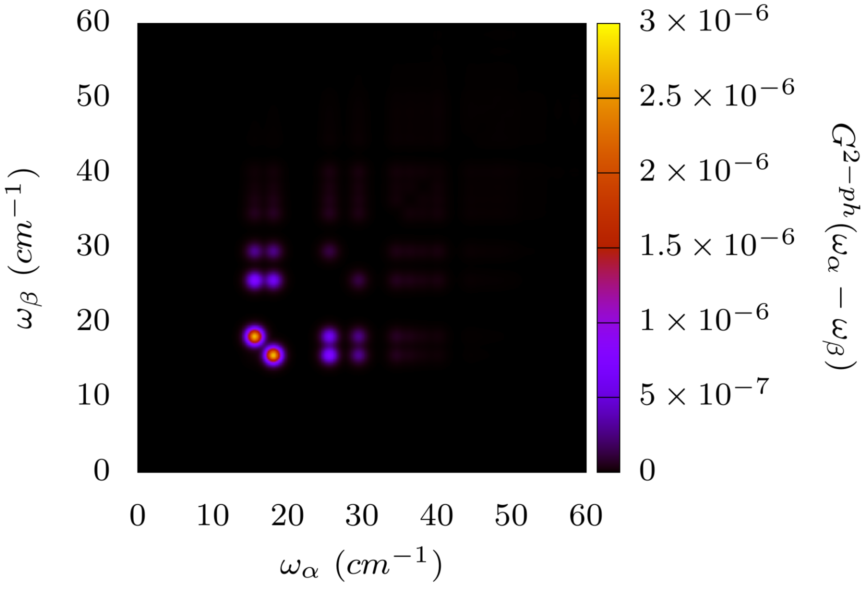

Fig. S5: Value of G^{2-ph} for the sole intra-Kramer doublet transition computed at $T = 5K$. The values of G^{2-ph} have been interpolated with a Gaussian smearing with $\sigma = 10$ cm^{-1}.

S2.3 Electrostatic modelling of spin-phonon coupling.

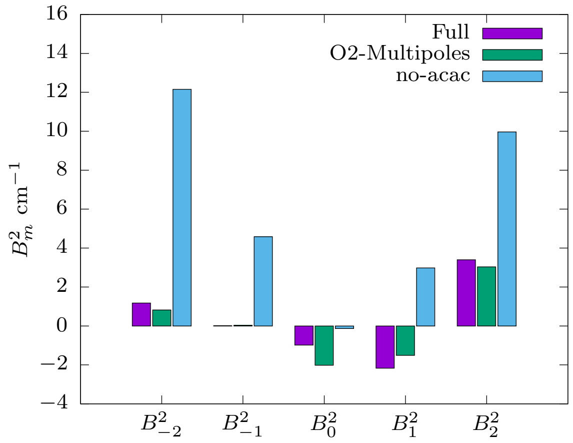

Fig. S6: **Crystal Field Shift.** The $l = 2$ Crystal Field parameters of Dyacac are plotted for the full model, including all the atoms explicitly (Full), the model where one acac ligand is replaced by the sole multipole expansion of the O-donor atoms (O2-Multipoles), and the model where one acac ligand is removed entirely (no-acac).

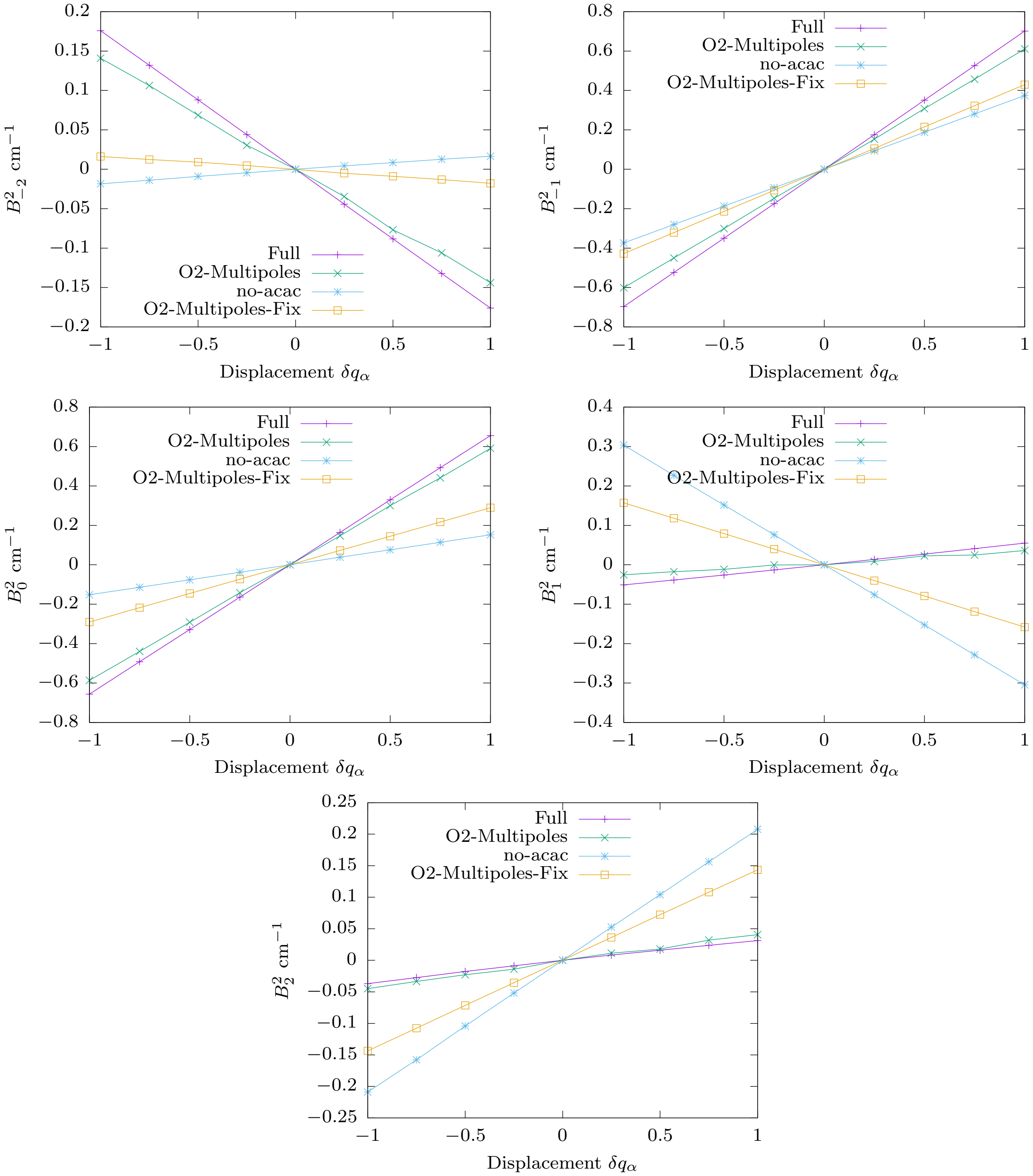

Fig. S7: **Crystal Field variation for vib1.** The $l = 2$ Crystal Field parameters of Dyacac are plotted along the molecular distortion associated to **vib1** for four different models: one including all the atoms explicitly (Full), one model where one acac ligand is replaced by the sole multipole expansion of the O-donor atoms (O2-Multipoles), one model where one acac ligand is removed entirely (no-acac), and one model where the acac's O-donor atom's multipolar expansion is kept constat to the equilibrium value (O2-Multipoles-Fix). The displacement is expressed in units of normal mode.

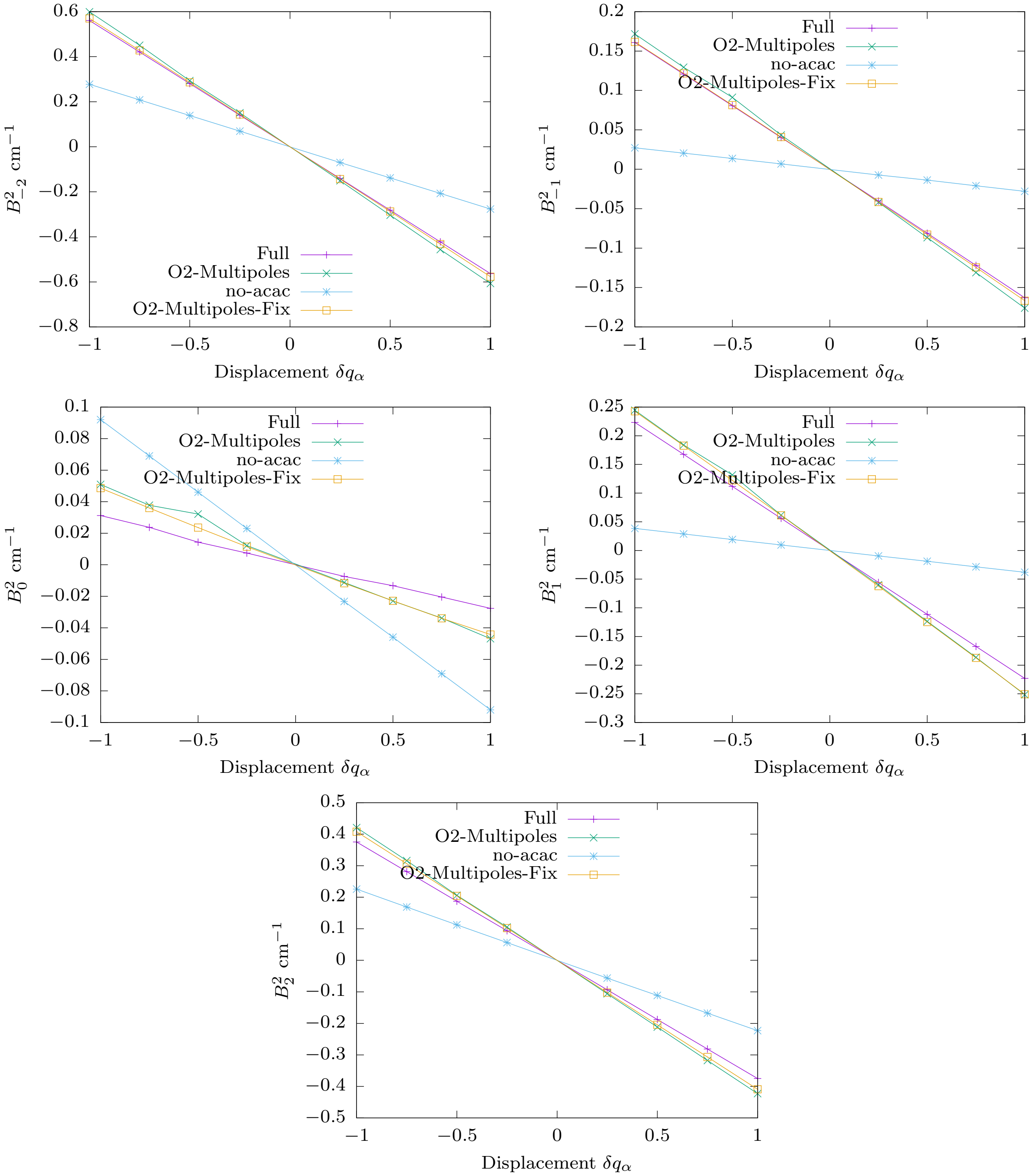

Fig. S8: **Crystal Field variation for vib2.** The $l = 2$ Crystal Field parameters of Dyacac are plotted along the molecular distortion associated to **vib2** for four different models: one including all the atoms explicitly (Full), one model where one acac ligand is replaced by the sole multipole expansion of the O-donor atoms (O2-Multipoles), one model where one acac ligand is removed entirely (no-Acac), and one model where the acac's O-donor atom's multipolar expansion is kept constat to the equilibrium value (O2-Multipoles-Fix). The displacement is expressed in units of normal mode.

S2.4 Derivatives of Electric Dipoles

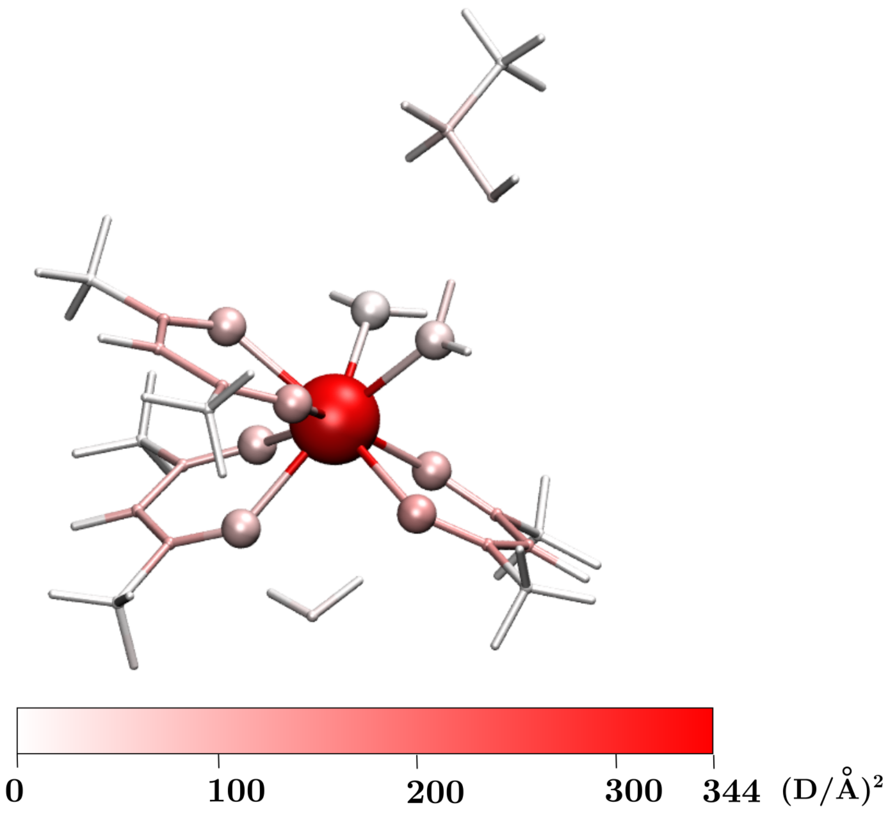

Fig. S9: Magnitude of the first derivative of electric dipole resolved by atomic site.

In the main text the same image was shown (Fig. 5D) but the value of the first derivative for Dy ion was artificially set to zero. The reason why the lanthanide ion shows a lower value of coupling, with respect to the one estimated from the dipole moment derivative, is connected to symmetry arguments. Due to its position at the centre of the complex, Cartesian displacements of the ion can be approximately be seen as anti-symmetric. This type of distortion is less couples to the spin and will therefore present a small derivative. On the other hand, anti-symmetric displacements are those more strongly contributing to the dipole moment derivatives. Cartesian displacements of atoms belonging to the ligands have a much less pronounced symmetry and these arguments do not apply to them, making

the correlation between dipole moment derivatives and spin-phonon coupling arise without interference.

In order to explain why anti-symmetric displacements do not lead to large spin-phonon coupling, let us take for simplicity two point charges at the opposite sides of a Ln ion. In the case of a charge approaching and an other one withdrawing from the ion, i.e. an anti-symmetric stretching, the two contributions to the spin-phonon will tend to cancel each other, due to the fact that their effect is the same in magnitude but reverse in sign. The opposite would happen for a symmetric stretching, where the effect of the two identical charges sum up.

S2.5 Convergence of relaxation time.

Relaxation time is computed by approximating the Dirac delta functions appearing in Eqs. 4-6 of the main text with a Gaussian function with smearing σ

$$\frac{1}{\sigma\sqrt{\pi}} e^{-\frac{\omega^2}{\sigma^2}} \xrightarrow{\sigma \to 0} \delta(\omega) \, . \tag{S5}$$

The left and right panels of Fig. S10 show the dependence of the Orbach and Raman computed relaxation times, respectively, as a function of σ. It is possible to note that τ converges to a value for all temperatures as σ increases. A rigorous estimation of the harmonic limit expressed by Eq. S5 can only be obtained by converging τ with respect to the number of reciprocal space **q**-points.[10] However, in a previous work,[24] it was indicated that a good approximation of this limit can be obtained increasing the size of σ and only including Γ-point phonons. This is probably a consequence of the large number of atoms in the unit cell of this class of compounds, which already offers a fine sampling of the vibrational density of states at the energy of the first optical phonons. However, this is not expected to hold for the gas-phase calculation of molecular vibrations.

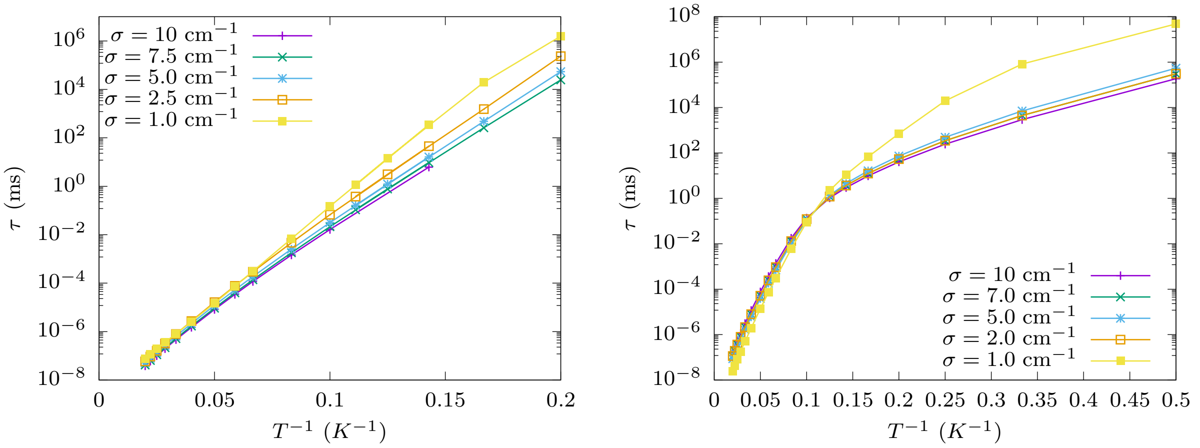

Fig. S10: Dependence of relaxation time with respect to the Gaussian smearing. Top left and right panels reports the dependence of the Orbach and Raman relaxation time computed as function of a Gaussian smearing, respectively.

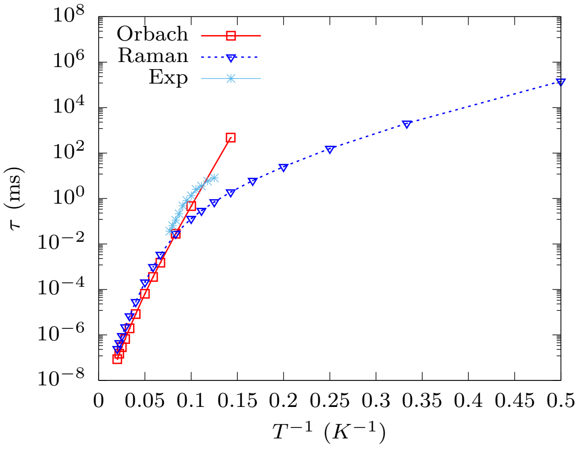

Fig. S11: **Relaxation rate with no effect of the Madelung potential.** The Orbach and Raman relaxation rate is computed without including the effect of the electrostatic field of the point charges on the static Crystal Field Hamiltonian.

S2.6 Effect of anharmonic phonons.

The derivation of Eqs. 3 of the main manuscript passes through the integration of the phonon correlation time function, G^{1-ph}

$$G^{1-ph}(t) = \int_0^{\infty} q_\alpha(t) q_\alpha(0) e^{i\omega t} dt \,. \tag{S6}$$

If the phonons are assumed to perfectly harmonic, the integral converges to

$$G^{1-ph} = n_\alpha \delta(\omega - \omega_\alpha) + (n_\alpha + 1)\delta(\omega + \omega_\alpha) \,. \tag{S7}$$

However, if phonon-phonon dissipation is present through anharmonic interactions, the phonons are exponentially damped. this is expressed by the ω_α assuming a complex value, where the immaginary component corresponds to the inverse of the q_α mode's lifetime, Δ_α. In such a scenario, the Dirac deltas appearing in Eq. S7 are replaced by Lorentzian functions

$$G^{1-ph} = n_\alpha \frac{\Delta_\alpha}{\Delta_\alpha^2 + (\omega - \omega_\alpha)^2} + (n_\alpha + 1) \frac{\Delta_\alpha}{\Delta_\alpha^2 + (\omega + \omega_\alpha)^2} \,. \tag{S8}$$

The effect of applying a constant Lorentzian smearing of Eq. S8 in the simulation of Orbach and Raman relaxation time is reported in the top right and top left panels of Fig. S12, respectively. It is shown that Orbach relaxation is strongly affected in the low temperature regime, where out-of-resonance low-energy phonons are able to induce a transition to the excited KD thanks to the long tail of their Lorentzian-smeared linewidth. However, from $T > 10$ K, resonant phonons are enough populated to drive relaxation and the anharmonic Orbach relaxation time becomes qualitatively identical to the harmonic case estimated with a Gaussian function.[24]

In addition to a constant Δ function, it is also possible to model the T-dependency of

the phonon lifetime as suggested by Lunghi A. *et al.*[34]

$$\Delta_T = \Delta + \omega_\alpha \frac{e^{\hbar\omega_\alpha/2K_bT}}{(e^{\hbar\omega_\alpha/K_bT} - 1)} \tag{S9}$$

The results obtained employing this model for different values of the constant offset Δ are reported in the bottom-left panel of Fig. S12. Besides a slight change in the slope of τ vs T for $T < 10$ K, the results are qualitatively close to those obtained with a constant Lorentzian smearing. Finally, the bottom-right panel of Fig. S12 reports the effect of applying the same expression used for Δ_T, with $\Delta = 0.0$ cm^{-1}, to a Gaussian smearing, σ_T. We remark that this does not represent an appropriate description of the homogeneous broadening due to vibrational anharmonicity. The Gaussian function, going to zero very quickly, does not reproduce the effect of the long tails of the Lorentzian function and results in non-physical results of infinite spin lifetime. However, this result is a consequence of the limited sampling of the Brillouin zone phonons. A correct integration of the vibrational Brillouin zone, as explained by Lunghi *et al.*,[10,24,35] would lead back to the harmonic limit. The latter is expected to be very close to the values obtained with a finite-size σ, as detailed in the previous section.

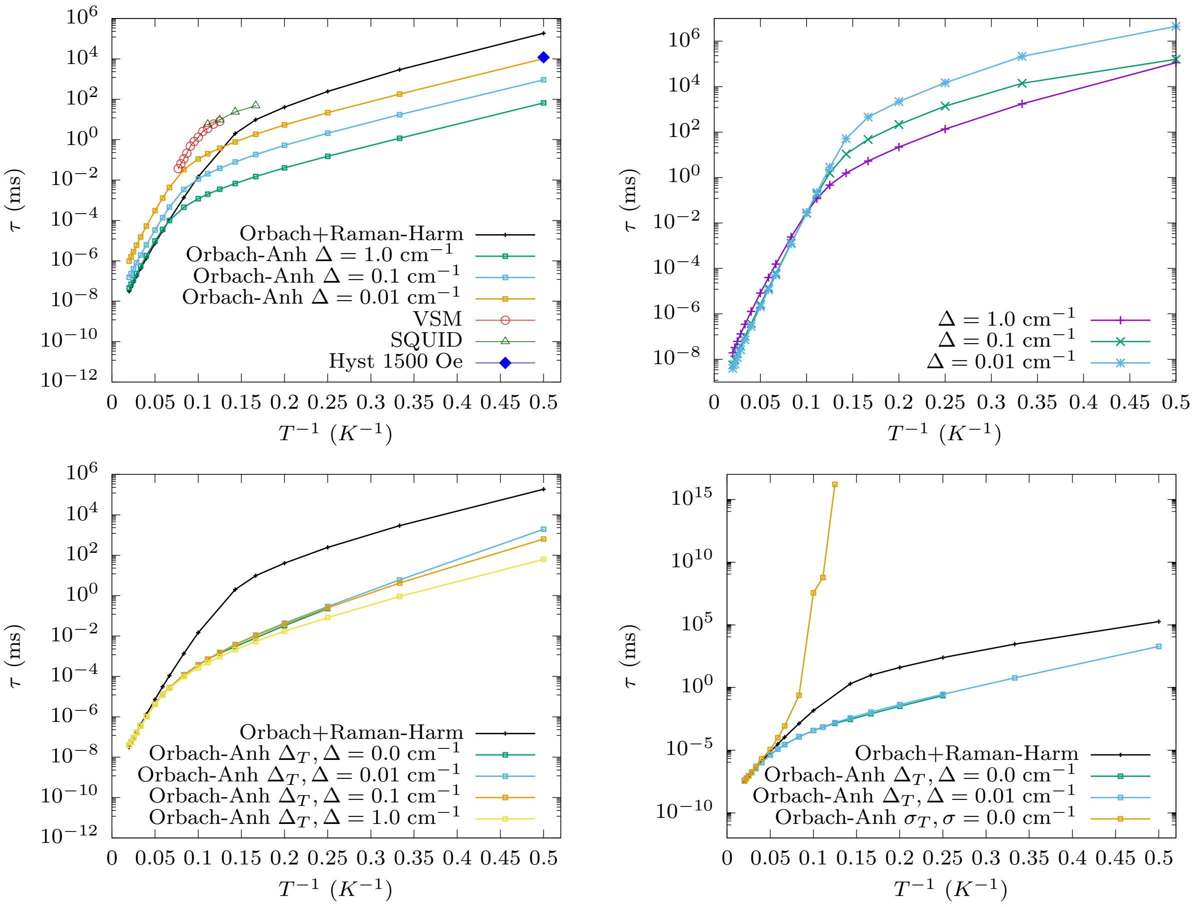

Fig. S12: **Effect of anharmonic phonons on the relaxation time.** The top-left panel reports a comparison between the results obtained with harmonic phonons, for both Orbach and Raman mechanisms, and the results obtained with the sole Orbach in the presence of anharmonic phonons with a constant Lorentzian linewidth Δ. The top-right panel describes the dependence of the sole Raman relaxation in the presence of anharmonic phonons with a constant Lorentzian linewidth Δ. The bottom left panel shows the effect of a T-dependent Lorentzian linewidth proposed by Lunghi *et al*,[34] Δ_T, with and without the addition of a constant offset Δ, whose value is reported in the key. The bottom right panel shows the results of applying the T-dependent linewidth proposed by Lunghi A. *et al.*[34] to a Gaussian smearing, σ_T.

S2.7 DFT Calculations on Isolated Ligands

Density Functional Theory calculations were performed with ORCA[36] to compute mulliken charges as a function of the CO bond length in three compounds: acetylacetonate (acac), acetone and 2-propoxide. B3LYP functional[37] and def2-TZVP basis sets[38] have been employed. The three structures have been optimized, then the CO bond lengths have been stretched and shortened of 0.05 $\AA$. The results are reported in Fig. S13. For acac molecule the two CO groups have been stretched and lengthened in opposite directions to mimick the antisymmetric CO stretching, as observed in vib1, and only the charge of one of two oxygens has been reported.

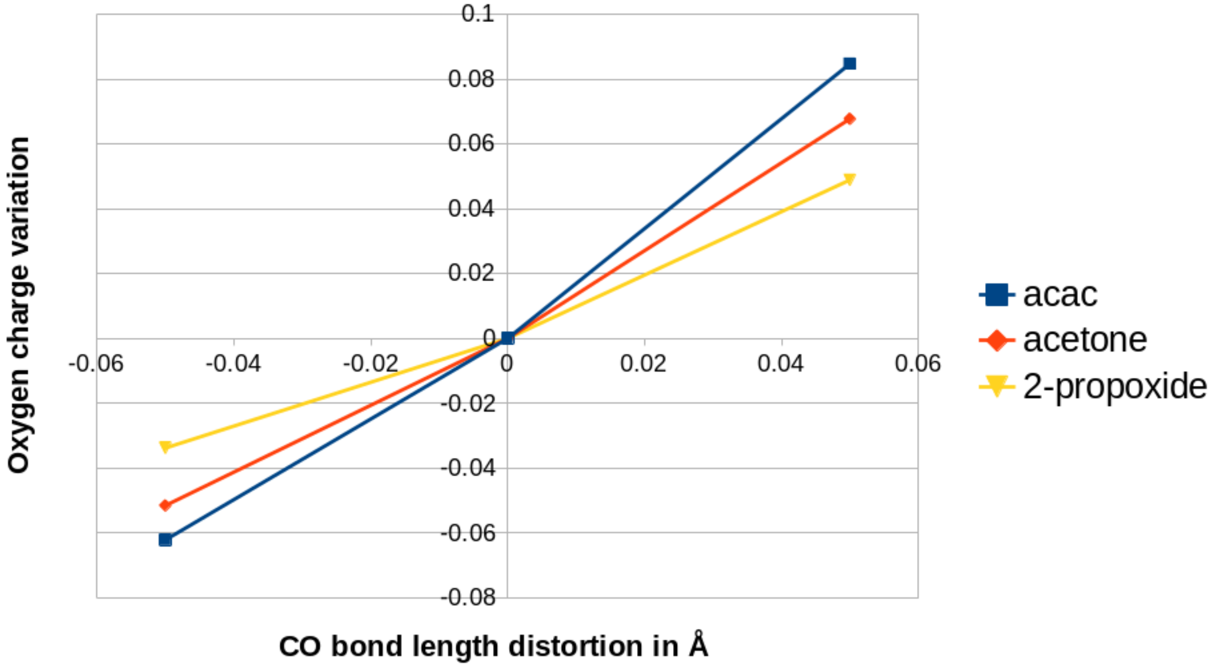

Fig. S13: Variations of the oxygen's Mulliken charge with respect to the equilibrium one, as a function of the CO bond length distortion.

S3 Experimental Details

Synthesis and characterization. All the employed chemicals and solvents were of reagent grade and used as such, without further purifications. All reactions have been conducted under atmospheric conditions. Compounds **Dyacac** and **Dy$_{0.1}$acac** were obtained with a reported strategy.[25] $Dy(NO_3)_3{\cdot}6H_2O$ (1 eq) was added to a hot solution of acetylacetone (3 eq) and KOH (3 eq) in ethanol, previously refluxed for 15 minutes. After adding the dysprosium precursor, a solid white precipitate formed out from the solution, and the resulting mixture is refluxed overnight with stirring. Once reflux was stopped, 15 mL of water was added to the mixture, and it was kept refluxing for additional 2 hours. After reaction completion, the mixture was cooled slowly to room temperature and then put in a freezer overnight, allowing the crystallization of the undesired byproduct identified as KNO3, which was removed by filtration. Evaporation of ethanol from solvents mixture yielded the desired product as prism-shaped crystals over two weeks. The diamagnetically diluted compound **Dy$_{0.1}$acac** has been obtained reacting a stoichiometric amount of $YNO_3{\cdot}6H2O$ and $DyNO_3{\cdot}6H2O$ and adopting the procedure above. The goodness of products was checked by powder X-Ray diffraction technique (PXRD), comparing experimental results and simulations obtained from the reported structure[25] (Figs. S28 and S29). The concentration of Dy^{3+} in **Dy$_{0.1}$acac** was confirmed both by ICP and AC susceptibility measurements (for further details, see section Magnetic Measurements and Figure S21). It is essential to mention that crystalline products change their crystal phase upon solvent evaporation. Indeed, while drying, the crystals may lose ethanol crystallization molecule. This fact might alter both the crystal structure and perturb the molecular coordination geometry sensibly because of the lowering of the hydrogen bonds network between coordinated and uncoordinated molecules (Figure S15). The obtained crystals must be stored in a sealed flask and kept into the crystallization medium to preserve the desired crystal structure. The loss of ethanol involves the fading of the transparency of the crystals, followed by a color change from transparent to pale white.

Powder X-ray Crystallography. Powder X-ray diffraction (PXRD) patterns of **Dyacac** and **Dy$_{0.1}$acac** were recorded on a Bruker New D8 Advance DAVINCI diffractometer in a theta-theta configuration equipped with a linear detector. The scans were collected on freshly filtered and crushed crystals, within the range 5-50° (2θ) using CuKα radiation (λ = 1.5398 Å) with a step of 0.025° and an exposure time of 0.6 sec/deg. Obtained powder patterns are reported for **Dyacac** and **Dy$_{0.1}$acac** in Figs. S28 and S29, respectively. Simulated patterns were generated from the atomic coordinates of the single-crystal structure solutions[25] (CCDC-) using the Mercury CSD 4.0 software (copyright CCDC, http://www.ccdc.cam.ac.uk/mercury/) using a FWHM (full width at half maximum) of 0.1°, and a 2θ step of 0.025°. The alteration of crystallinity was followed by repeating the measurements at different times. Comparing experimental results and simulated spectra (Figs. S28 and S29), it appears that the change in crystallinity occurs 12 minutes after the first exposition to the air and proceeds within the next period.

Single-crystal X-ray. Single crystal diffraction measurements were performed on an Oxford Xcalibur PX Ultra-Onyx CCD diffractometer, using an Enhance Ultra X-ray graphite-monochromated Cu Kα radiation (λ = 1.540 Å). Crystals of appropriate dimensions were taken from the crystallization medium and rapidly immersed in a grease suitable for low-temperature operations. Crystals were supported on a small acetate paper and mounted on a modified goniometer head for indexing.

Magnetic Measurements. Alternate Current (AC) magnetic susceptibility measurements were performed using both a *Quantum Design Physical Property Measurement System* (PPMS) and a *Quantum Design Magnetic Property Measurement System* (MPMS) to reach a large set of working frequencies spanning from 1 Hz to 10000 Hz. AC susceptibility by applying an oscillating field of 1 mT (PPMS) and 0.3 mT (MPMS) in the temperature range 8–15 K at 0 mT or setting the external static magnetic field up to 50 mT (PPMS). Measurements were performed on randomly oriented powder samples of **Dy$_{0.1}$acac**, which were prepared

by rapidly filtrating the crystals (previously ground with a spatula without removing them from crystallization waters). Thus, the crystals were encapsulated in a weighted holder, and the whole apparatus introduced into the instrument at 160 K. The resulting data are reported in Figs. S21-S24, which show the real (χ') and imaginary (χ") components of the magnetic susceptibility as a function of the frequency. Susceptibility data were corrected for the sample holder previously measured using the same conditions, and for the diamagnetic contributions deduced by using Pascal's constant tables.[39] All the magnetic data were rescaled considering a concentration of magnetic species of about 10 %.

Fits of the imaginary component of the susceptibility have been implemented by adopting the following modified Debye model, which has been extended as sum of single contributions:

$$\chi'' = \frac{(\chi_{T,1} - \chi_{S,1})(\omega\tau_1)^{1-\alpha_1} cos(\frac{\pi\alpha_1}{2})}{1 + 2(\omega\tau_1)^{1-\alpha_1} sin(\frac{\alpha_1}{2}) + (\omega\tau_1)^{2-2\alpha_1}} + \frac{(\chi_{T,2} - \chi_{S,2})(\omega\tau_2)^{1-\alpha_2} cos(\frac{\pi\alpha_2}{2})}{1 + 2(\omega\tau_2)^{1-\alpha_2} sin(\frac{\alpha_2}{2}) + (\omega\tau_2)^{2-2\alpha_2}} \quad \text{(S10)}$$

Where 1 and 2 subscritps refer to the two contributions employed, $\chi_T - \chi_s$ is the difference between the isothermal and adiabatic susceptibility, τ is the relaxation time constant, and α accounts for the width of the relaxation times distribution.

Vibrating Sample Mode. Experiments were performed on the same sample that was previously measured in PPMS device. Hysteresis were recorded in a Oxford Instruments MAGLAB2000 cryostat using a vibrating sample magnetometer.

The relaxation time at 2 K has been estimated at 2 K and 1.5 T/min field rate by using the following equation (courtesy by Prof. Fernando Luis and based on a paper by Kurkijärvi[40]):

$$\tau = -\frac{-t_M}{ln\left[1 - \frac{|\Delta B|\left(\frac{dM_{up}}{dB} + \frac{dM_{down}}{dB}\right)}{M_{up} - M_{down}}\right]} \quad \text{(S11)}$$

where t_M is the measurement time that is related to the speed of the magnetic field sweep, ΔB is the magnetic field step, M_{up} and M_{down} the upper and lower hysteresis arms, respectively.

Cantilever Torque Magnetometry. Experiments were performed by using a home-made two-legged CuBe cantilever plate separated by 0.1 mm from a gold plate, which acts as a capacitor. The capacitance is detected with an Andeen-Hegerling 2500 A Ultra Precisio Capacitance Bridge. The cantilever is inserted into an Oxford Instruments MAGLAB2000 cryostat with a vertical oriented magnetic field. The rotation of the cantilever is automated around a fixed axis. Sample preparation consists of the indexing of a single-crystal by means of X-Ray diffraction (see above) to identify the crystallographic directions and refer them to the CTM laboratory reference frame. Due to the degradation of the sample, this procedure has been done as quick as possible (few minutes in total), while the indexing of the crystal has been carried out at N_2 temperatures. This resulted in an estimated uncertainty of the actual orientation of the crystal of about 5°. A refinement of the magnetic anisotropy orientation can be done by fitting the torque experimental data. However only minor variations in the main magnetic axes' orientations are observed, particularly regarding the magnetic easy axis. As a consequence of the large number of parameters, the *ab initio* crystal field parameters were kept fixed to the computed values, while the crystal field reference frame was left free to rotate. As it can be seen in Fig. S18, this procedure does not increase the already good agreement of simulations for Rot2, whereas it improves for Rot1. The angle among easy axes identified by simulations and fits is 10° (see Table S7), while a slightly larger gap can be found for the two axes with hard character (Figs. S18-S20). Simulations and fits were performed using a MATLAB code developed by Dr. M. Perfetti.[41]

Table S9: Orientation inside the molecular frame of the *ab initio* computed ground state g-tensor and the fitted anisotropy axes from CTM measurements

ab initio			
g_X	-0.790771	-0.197256	-0.579458
g_Y	-0.456903	-0.439741	0.773218
g_Z	-0.407333	0.876195	0.257608
CTM			
A_1	-0.6551	-0.2292	-0.7200
A_2	-0.6347	-0.3501	0.6889
A_3	-0.4099	0.9083	0.0839

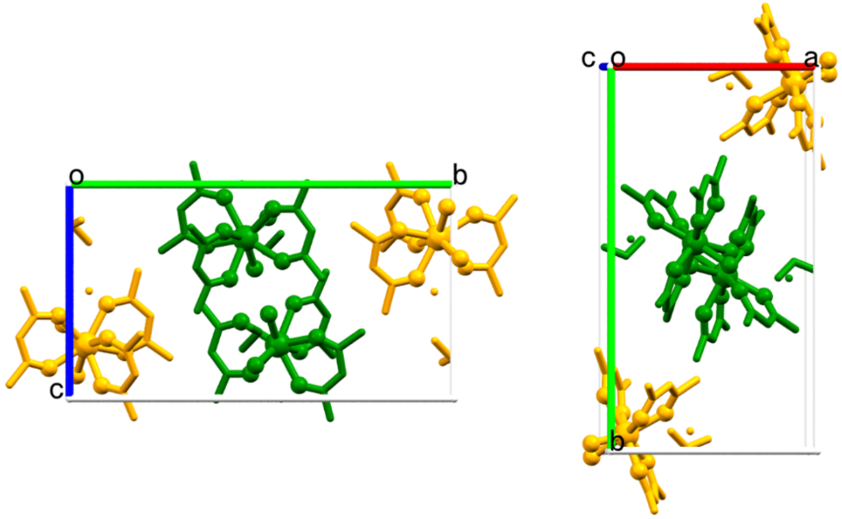

Fig. S14: Cartoon reporting the unit cells of the **Dyacac** structure extracted from the literature.[25] For the sake of clarity, hydrogen atoms have been removed from the molecular structures, and different molecules generated by inversion or glide plane symmetry operations are reported in green and orange, respectively.

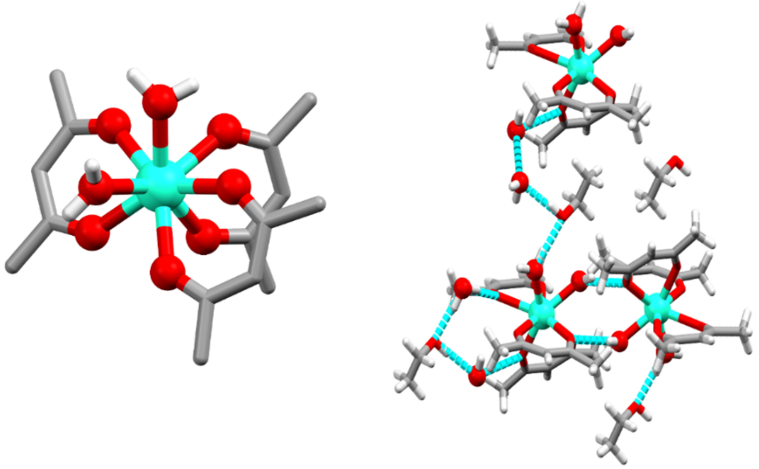

Fig. S15: Representations of $[Dy(acac)_2(H_2O)_2]\cdot EtOH\cdot H_2O$ extracted molecular structure. On the left side, a sketch of the single molecule is reported from the "top side", which best highlights the pseudo D_{2d} local symmetry. On the right side, it is reported the view on the molecular assembly which forms the crystal structure, in which hydrogen-bonds between different complexes and crystallization molecules are highlighted (cyan rods). Oxygen atoms of water molecules are represented as a sphere for clarity. Color code: Dy = cyan, O = red, C = grey, H = white.

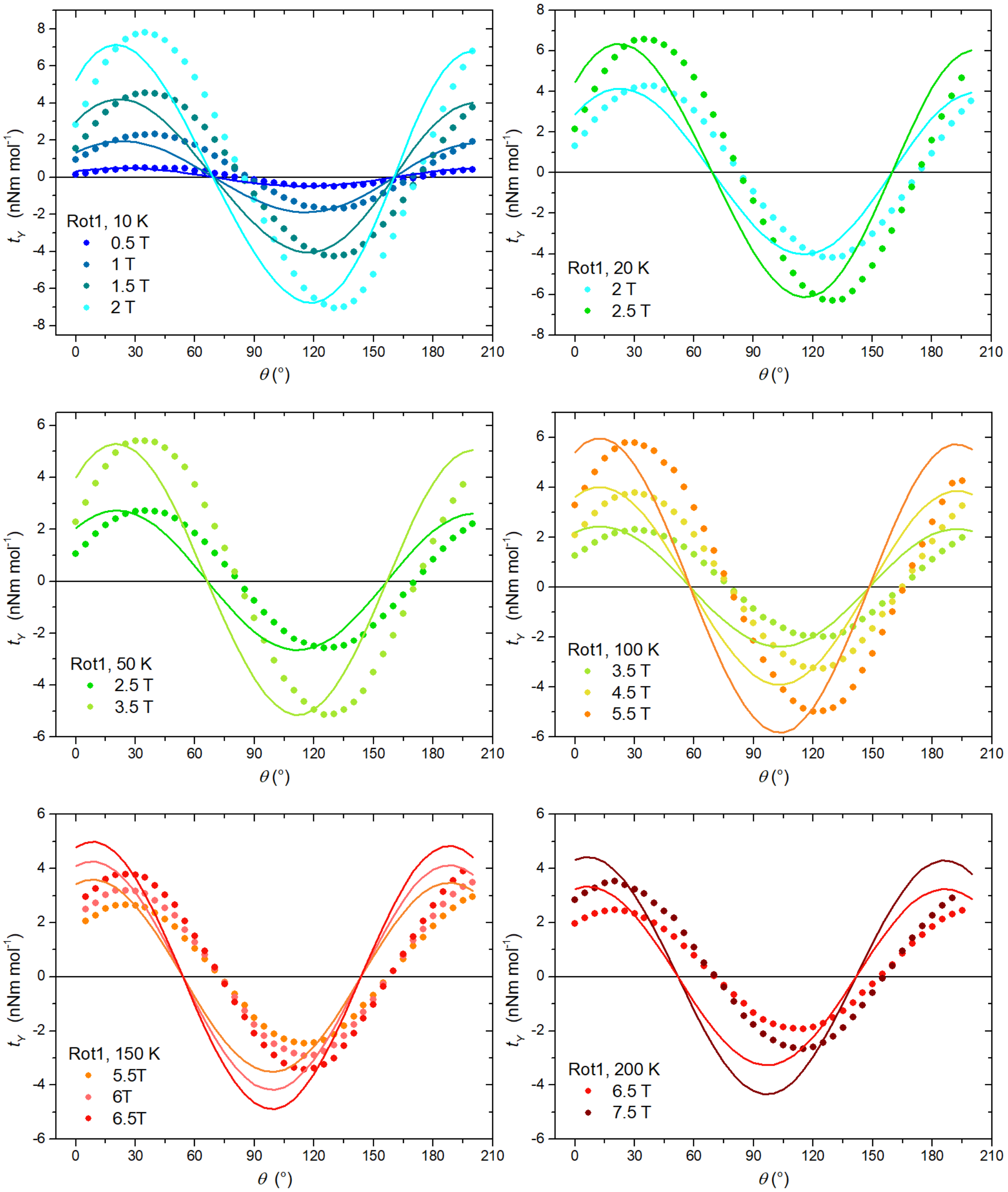

Fig. S16: **Dyacac** single-crystal torque magnetometry experiments (dot) and *ab initio* simulations (line) for Rot1 dataset at 10, 20, 50, 100, 150 and 200 K at different values of applied magnetic field.

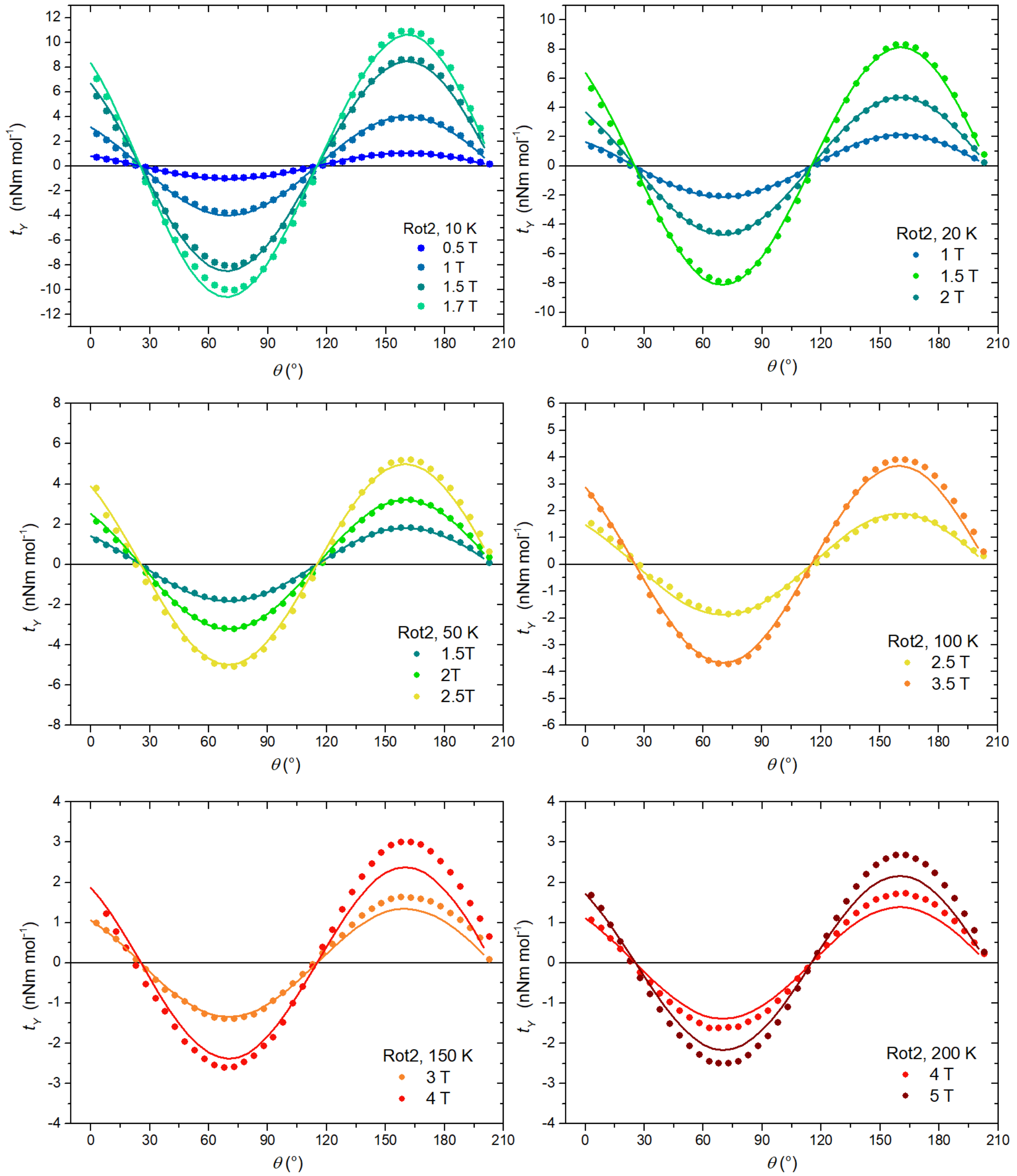

Fig. S17: **Dyacac** single-crystal torque magnetometry experiments (dot) and *ab initio* simulations (line) for Rot2 at 10, 20, 50, 100, 150 and 200 K at different values of applied magnetic field.

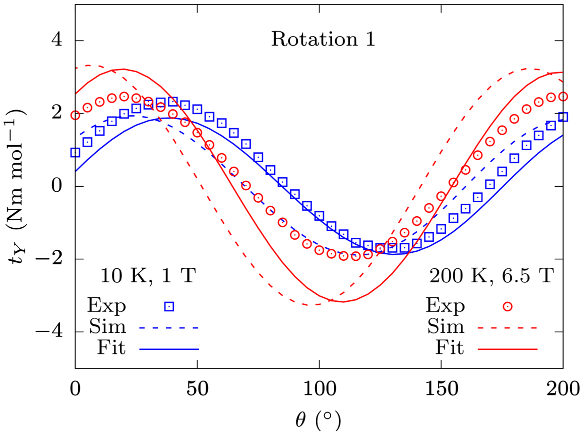

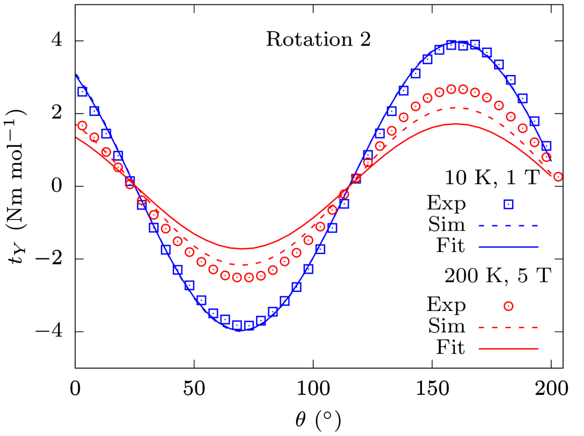

Fig. S18: **Magnetization torque measurement.** The torque momentum measured on **Dyacac** single crystal as a function of the rotation angle at 10 K (blue color and empty square symbols) and 200 K (red color and empty square symbols) for Rot1 (top left panel) and Rot2 (bottom left panel). The simulations are based on the parameters obtained by *ab initio* calculations. The fits are implemented by using the *ab initio* crystal field parameters and rotating the magnetic tensor.

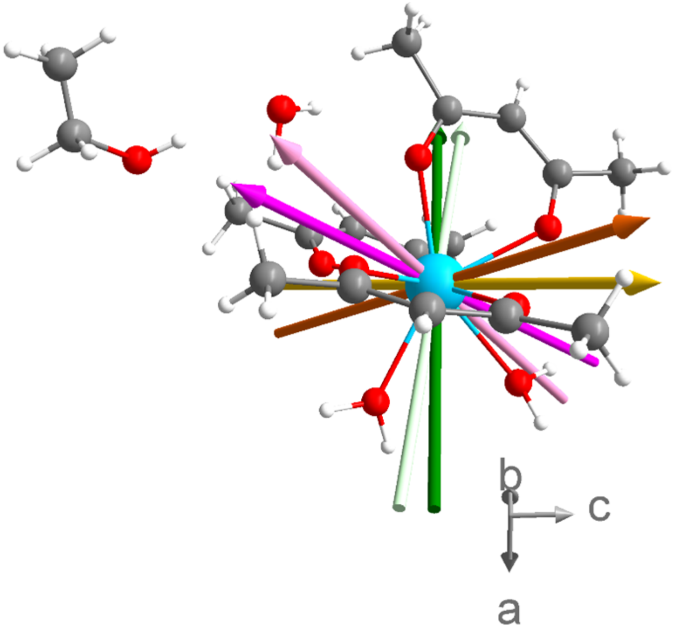

Fig. S19: **Dyacac** structure with simulated (light colours) and fitted (dark colours) principal magnetic axes viewed along b crystallographic direction.

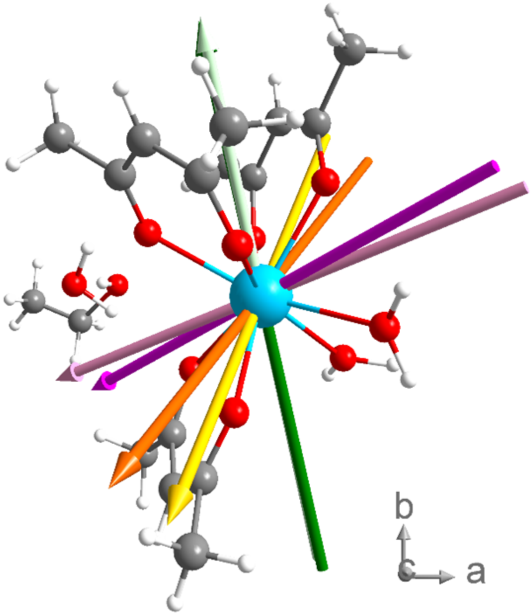

Fig. S20: **Dyacac** structure with simulated (light colours) and fitted (dark colours) principal magnetic axes viewed along $c*$ crystallographic direction.

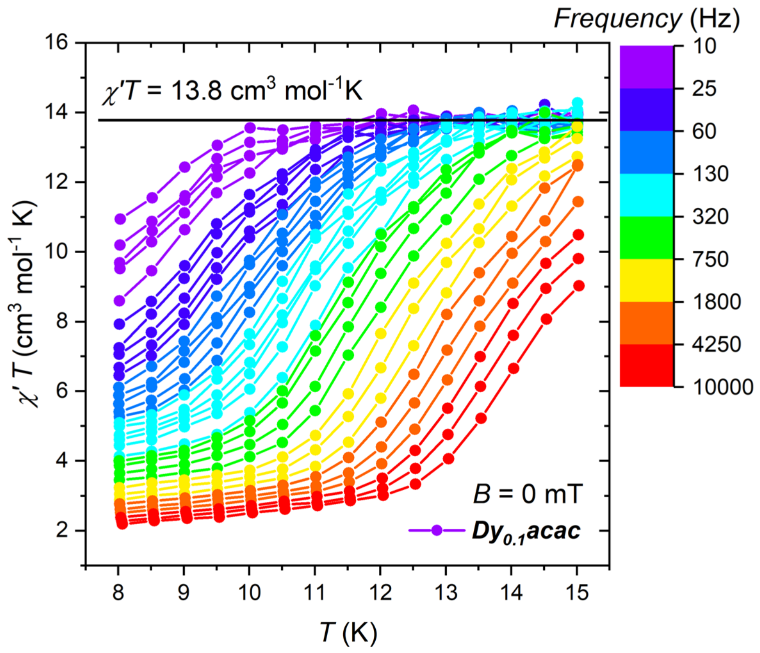

Fig. S21: Plot of the real component of the susceptibility reported as χ'T (cm^3 mol^{-1} K) as a function of T (K) at B = 0 mT. The experimental limit χ'T value at low frequency is highlighted in the Figure by the black line. The whole set of data has been rescaled considering a concentration of 10% of the total weight

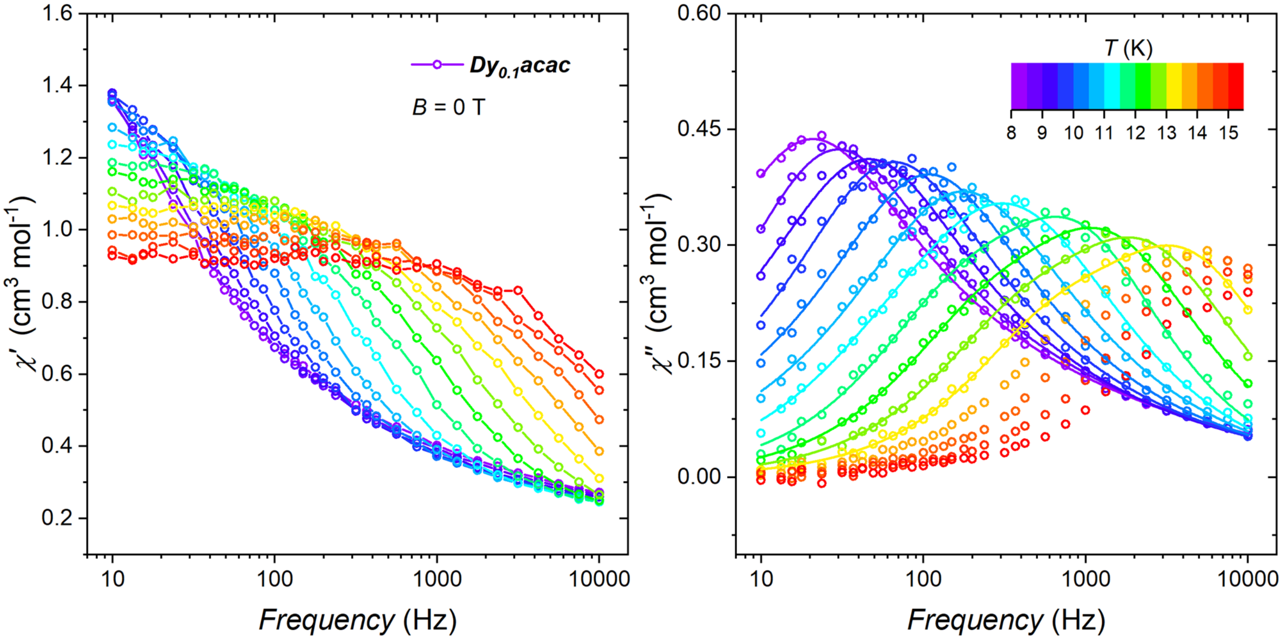

Fig. S22: Plots of χ' and χ" (cm^3 mol^{-1}) as a function of the frequency (Hz) in logarithmic scale, obtained from AC susceptibility measurements at different T (K) and at B = 0 mT on compound **Dy$_{0.1}$acac**. Experimental data are reported as dots. Curves resulting from the fitting of χ" based on Eq. S10 are represented as solid lines in the plot on the right, while solid lines in the plot of χ' are used as traces.

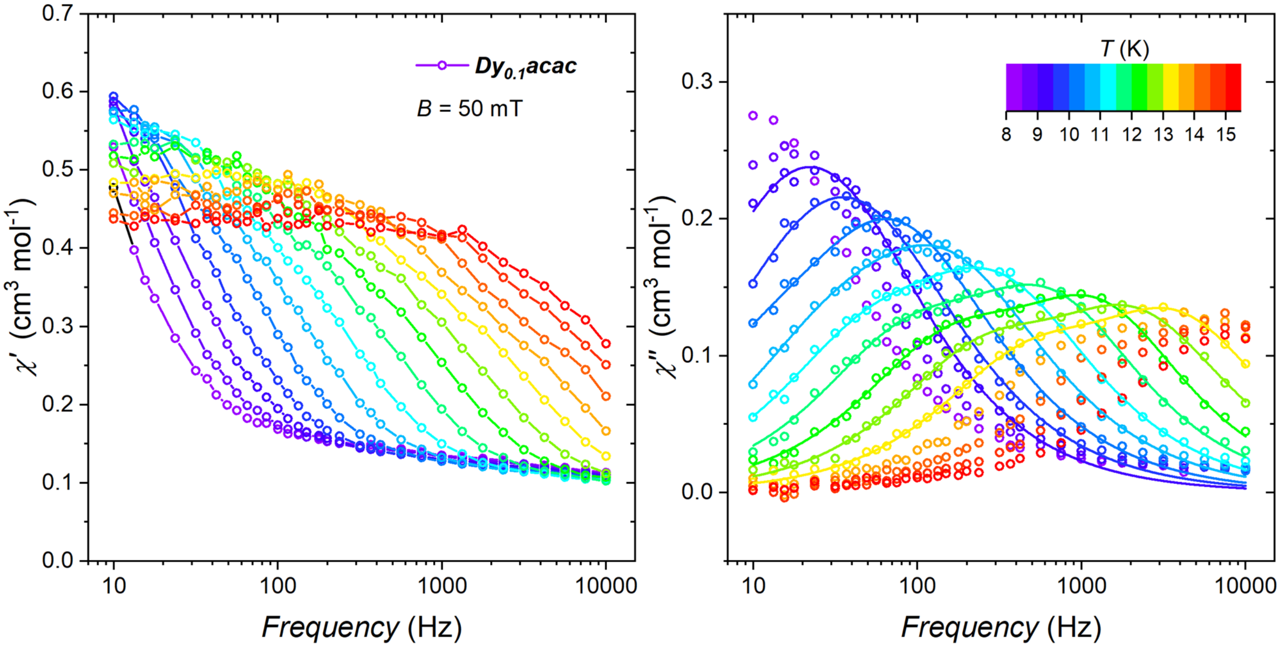

Fig. S23: Plots of χ' and χ" (cm^3 mol^{-1}) as a function of the frequency (Hz) in logarithmic scale, obtained from AC susceptibility measurements at different T (K) and at B = 50 mT on compound **Dy$_{0.1}$acac**. Experimental data are reported as dots. Curves resulting from the fitting of χ" based on Eq. S10 are represented as solid lines in the plot on the right, while solid lines in the plot of χ' are used as traces.

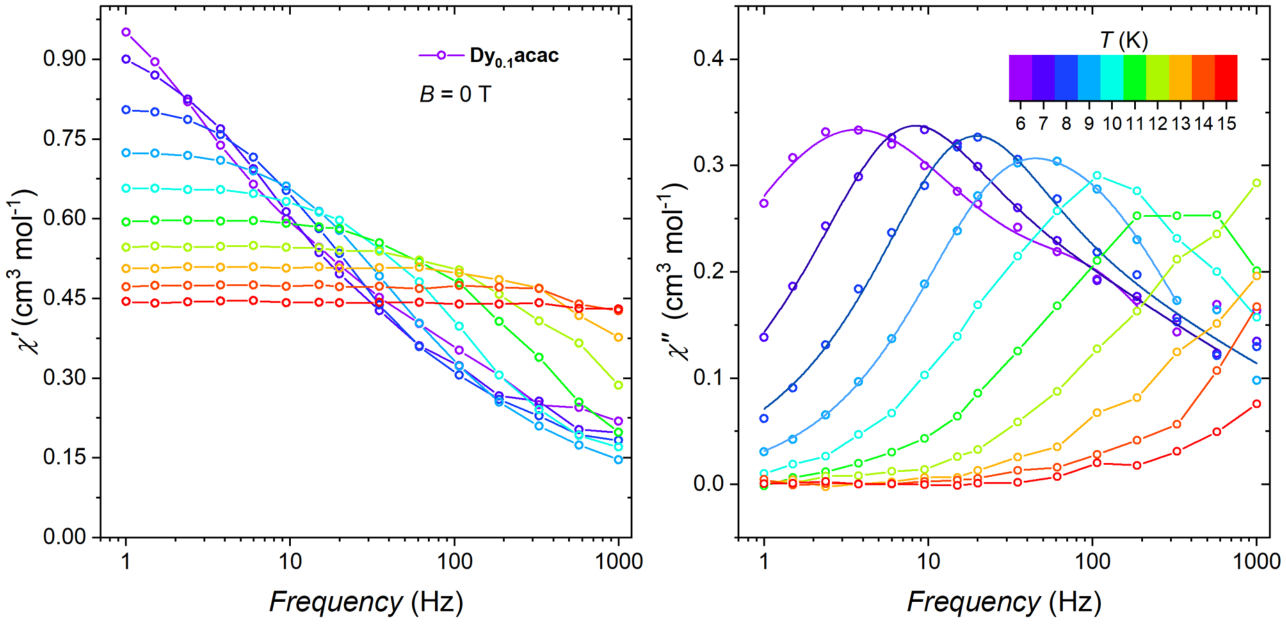

Fig. S24: Plots of χ' and χ" (cm^3 mol^{-1}) as a function of the frequency (Hz) in logarithmic scale, obtained from AC susceptibility measurements obtained from measurements on MPMS device at different T (K) and at B = 0 mT on compound **Dy$_{0.1}$acac**. Experimental data are reported as dots. Curves resulting from the fitting of χ" based on Eq. S10 are represented as solid lines in the plot on the right, while solid lines in the plot of χ' are used as traces.

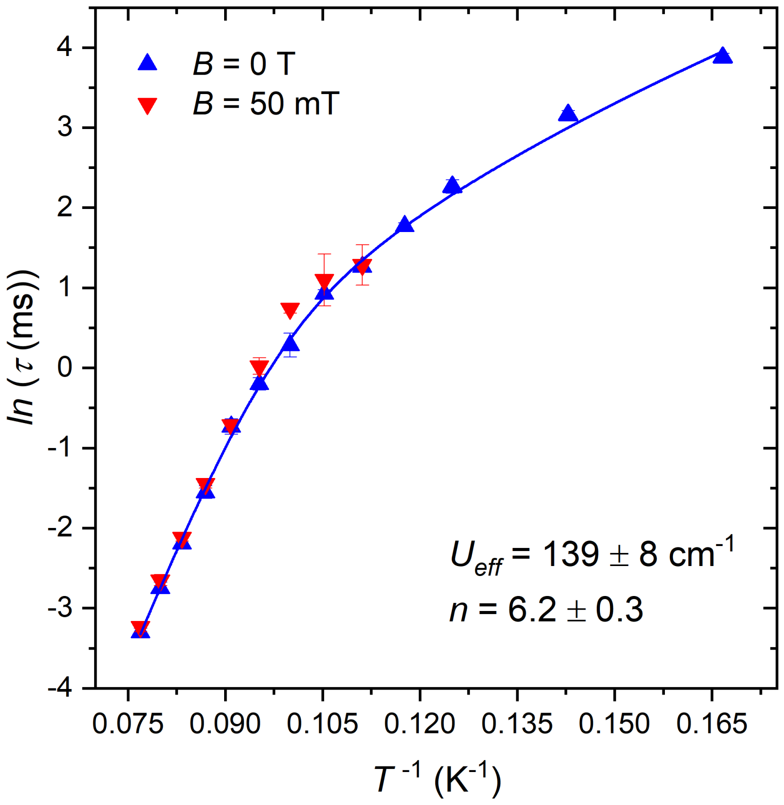

Fig. S25: Plot of τ values extracted from fits of AC data collected at 0 mT (Blue triangles) and 50 mT (red triangle), reported as ln(τ (ms)) vs. T^{-1} (K^{-1}). The continuous blue line represents the fit obtained by adopting the model:[29] $\tau^{-1} = CT^n + \tau_0^{-1}exp(\frac{U}{k_BT})$ $\tau_0^{-1} = (8.0 \pm 7.8) \cdot 10^{-9}ms^{-1}$, $U = (139 \pm 8)cm^{-1}$, $C = (3 \pm 2) \cdot 10^{-7}ms^{-1}K^{-n}$, $n = 6.2 \pm 0.3$.

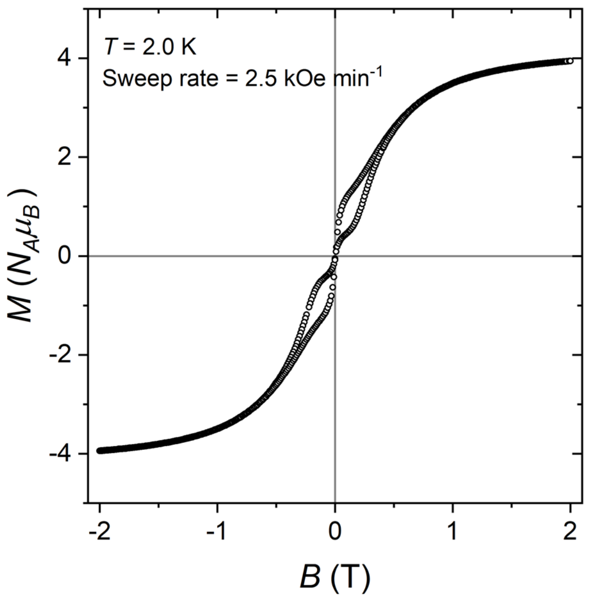

Fig. S26: Hysteresis loop recorded at 2 K on a $\mathbf{Dy_{0.1}acac}$ with a sweep rate of 2.5 kOe min^{-1}.

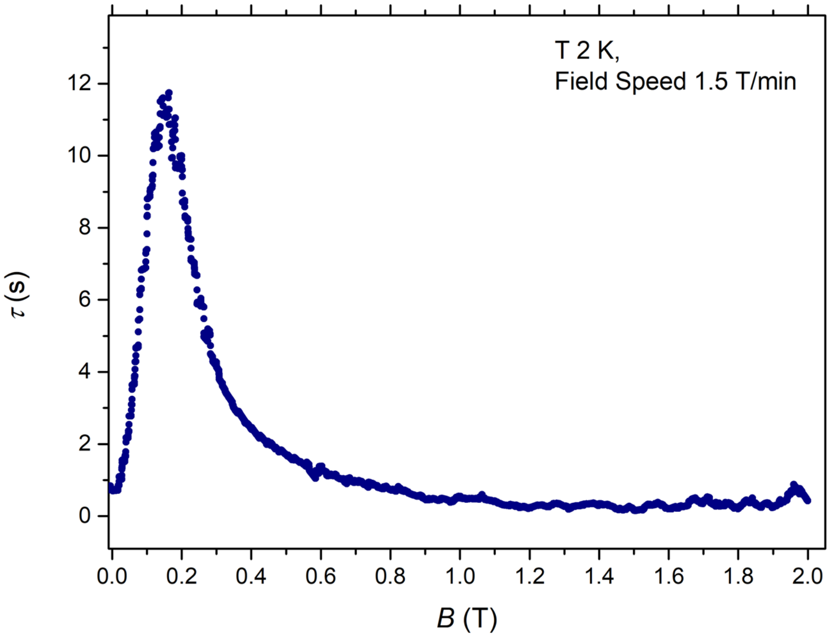

Fig. S27: Relaxation time extracted from the hysteresis measurement at 2 K with a sweep rate of 1.5 T min^{-1}.

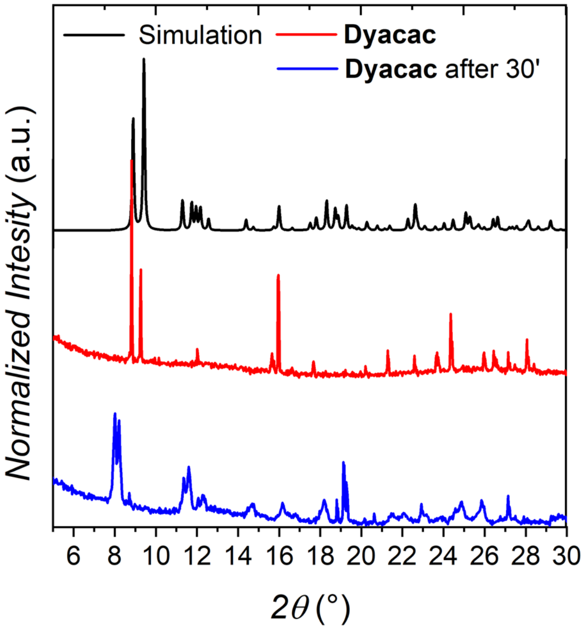

Fig. S28: Powder X-ray diffraction pattern obtained on a freshly filtered sample of **Dyacac** (red line). The experimental pattern is compared with the simulated one (black line),[25] and with the one obtained on a sample left to dry in air for 30 minutes (blue line).

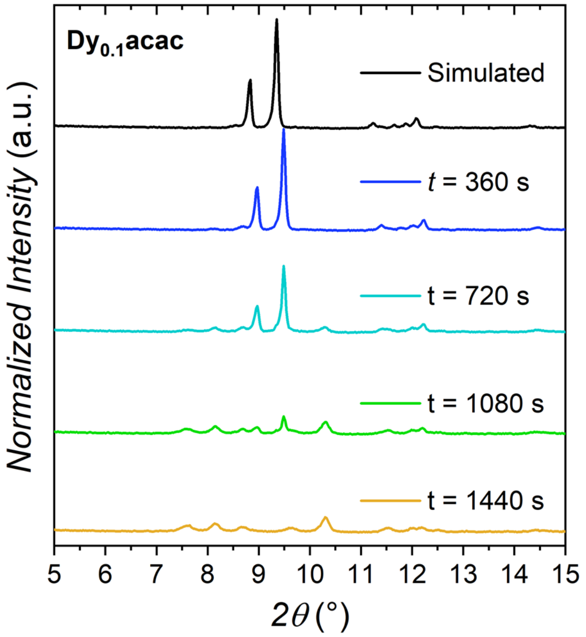

Fig. S29: Powder X-ray diffraction pattern collected between 5 and 15° on a freshly filtered sample of **Dy$_{0.1}$acac** (blue line). Each collection has a time length of 6 minutes, and the experiment has been repeated four times. All the obtained plots (from blue to yellow lines) show the change in the sample's crystallinity. The black line shows the simulation obtained from the structure of **Dyacac** reported in the literature[25]

References

(1) Kühne, T. D. et al. CP2K: An electronic structure and molecular dynamics software package - Quickstep: Efficient and accurate electronic structure calculations. *J. Chem. Phys.* **2020**, *152*, 194103.

(2) VandeVondele, J.; Krack, M.; Mohamed, F.; Parrinello, M.; Chassaing, T.; Hutter, J. Quickstep: Fast and accurate density functional calculations using a mixed Gaussian and plane waves approach. *Comput. Phys. Commun.* **2005**, *167*, 103–128.

(3) Goedecker, S.; Teter, M.; Hutter, J. Separable Dual-Space Gaussian Pseudopotentials. *Phys. Rev. B* **1996**, *54*, 1703–1710.

(4) Hartwigsen, C.; Goedecker, S.; Hutter, J. Relativistic separable dual-space Gaussian pseudopotentials from H to Rn. *Phys. Rev. B* **1998**, *58*, 3641–3662.

(5) Krack, M. Pseudopotentials for H to Kr optimized for gradient-corrected exchange-correlation functionals. *Theor. Chem. Acc.* **2005**, *114*, 145–152.

(6) Perdew, J. P.; Burke, K.; Ernzerhof, M. Generalized Gradient Approximation Made Simple. *Phys. Rev. Lett.* **1996**, *77*, 3865–3868.

(7) Grimme, S. Semiempirical GGA-type density functional constructed with a long-range dispersion correction. *J. Comput. Chem.* **2006**, *27*, 1787–1799.

(8) Grimme, S.; Antony, J.; Ehrlich, S.; Krieg, H. A consistent and accurate ab initio parametrization of density functional dispersion correction (DFT-D) for the 94 elements H-Pu. *J. Chem. Phys.* **2010**, *132*, 154104.

(9) Grimme, S.; Ehrlich, S.; Goerigk, L. Effect of the damping function in dispersion corrected density functional theory. *J. Comput. Chem.* **2011**, *32*, 1456–1465.

(10) Lunghi, A.; Sanvito, S. How do phonons relax molecular spins? *Sci. Adv.* **2019**, *5*, eaax7163.

(11) Malmqvist, P.-Å.; Rendell, A.; Roos, B. O. The Restricted Active Space Self-Consistent-Field Method, Implemented with a Split Graph Unitary Group Approach. *J. Phys. Chem.* **1990**, *94*, 5477–5482.

(12) Roos, B. O.; Malmqvist, P.-[U+FFFD]. *Phys. Chem. Chem. Phys.* **2004**, *6*, 2919.

(13) Malmqvist, P. Å.; Roos, B. O.; Schimmelpfennig, B. The restricted active space (RAS) state interaction approach with spin–orbit coupling. *Chem. Phys. Lett.* **2002**, *357*, 230–240.

(14) Aquilante, F. et al. <scp>Molcas</scp> 8: New capabilities for multiconfigurational quantum chemical calculations across the periodic table. *J. Comput. Chem.* **2016**, *37*, 506–541.

(15) Aquilante, F. et al. Modern quantum chemistry with [Open]Molcas. *J. Chem. Phys.* **2020**, *152*, 214117.

(16) Chibotaru, L. F. *Adv. Chem. Phys.*; 2013; pp 397–519.

(17) Ungur, L.; Chibotaru, L. F. Ab Initio Crystal Field for Lanthanides. *Chem. - A Eur. J.* **2017**, *23*, 3708–3718.

(18) Gagliardi, L.; Lindh, R.; Karlstrom, G. Local properties of quantum chemical systems: The LoProp approach. *J. Chem. Phys.* **2004**, *121*, 4494.

(19) Roos, B. O.; Lindh, R.; Malmqvist, P. k.; Veryazov, V.; Widmark, P. O. Main Group Atoms and Dimers Studied with a New Relativistic ANO Basis Set. *J. Phys. Chem. A* **2004**, *108*, 2851–2858.

(20) Roos, B. O.; Lindh, R.; Malmqvist, P.-Å.; Veryazov, V.; Widmark, P.-O.; Borin, A. C. New Relativistic Atomic Natural Orbital Basis Sets for Lanthanide Atoms with Applications to the Ce Diatom and LuF 3. *J. Phys. Chem. A* **2008**, *112*, 11431–11435.

(21) Briganti, M.; Garcia, G. F.; Jung, J.; Sessoli, R.; Le Guennic, B.; Totti, F. Covalency and magnetic anisotropy in lanthanide single molecule magnets: the DyDOTA archetype. *Chem. Sci.* **2019**, *10*, 7233–7245.

(22) Blöchl, P. E. Electrostatic decoupling of periodic images of plane-wave-expanded densities and derived atomic point charges. *J. Chem. Phys.* **1995**, *103*, 7422–7428.

(23) Lunghi, A.; Totti, F.; Sanvito, S.; Sessoli, R. Intra-molecular origin of the spin-phonon coupling in slow-relaxing molecular magnets. *Chem. Sci.* **2017**, *8*, 6051–6059.

(24) Lunghi, A.; Sanvito, S. Multiple spin–phonon relaxation pathways in a Kramer single-ion magnet. *J. Chem. Phys.* **2020**, *153*, 174113.

(25) Jiang, S.-D.; Wang, B.-W.; Su, G.; Wang, Z.-M.; Gao, S. A Mononuclear Dysprosium Complex Featuring Single-Molecule-Magnet Behavior. *Angew. Chemie Int. Ed.* **2010**, *49*, 7448–7451.

(26) ALVAREZ, S.; ALEMANY, P.; CASANOVA, D.; CIRERA, J.; LLUNELL, M.; AVNIR, D. Shape maps and polyhedral interconversion paths in transition metal chemistry. *Coord. Chem. Rev.* **2005**, *249*, 1693–1708.

(27) Cole, K. S.; Cole, R. H. Dispersion and Absorption in Dielectrics I. Alternating Current Characteristics. *J. Chem. Phys.* **1941**, *9*, 341–351.

(28) Dekker, C.; Arts, A. F. M.; de Wijn, H. W.; van Duyneveldt, A. J.; Mydosh, J. A. Activated dynamics in a two-dimensional Ising spin glass: Rb2Cu1-xCoxF4. *Phys. Rev. B* **1989**, *40*, 11243–11251.

(29) Liddle, S. T.; van Slageren, J. Improving f-element single molecule magnets. *Chem. Soc. Rev.* **2015**, *44*, 6655–6669.

(30) Tennant, W. C.; Walsby, C. J.; Claridge, R. F. C.; McGavin, D. G.; Tennant, W. C.; Claridge, R. F. C. Rotation matrix elements and further decomposition functions of two-

vector tesseral spherical tensor operators; their uses in electron paramagnetic resonance spectroscopy. *J. Phys. Condens. Matter* **2000**, *12*, 9481–9495.

(31) Maurice, R.; Bastardis, R.; Graaf, C. d.; Suaud, N.; Mallah, T.; Guihéry, N. Universal Theoretical Approach to Extract Anisotropic Spin Hamiltonians. *J. Chem. Theory Comput.* **2009**, *5*, 2977–2984.

(32) Chibotaru, L. F.; Ungur, L. Ab initio calculation of anisotropic magnetic properties of complexes. I. Unique definition of pseudospin Hamiltonians and their derivation. *J. Chem. Phys.* **2012**, *137*, 064112.

(33) Jung, J.; Islam, M. A.; Pecoraro, V. L.; Mallah, T.; Berthon, C.; Bolvin, H. Derivation of Lanthanide Series Crystal Field Parameters From First Principles. *Chem. Eur. J.* **2019**, *25*, 15112–15122.

(34) Lunghi, A.; Totti, F.; Sessoli, R.; Sanvito, S. The role of anharmonic phonons in under-barrier spin relaxation of single molecule magnets. *Nat. Commun.* **2017**, *8*, 14620.

(35) Lunghi, A.; Sanvito, S. The Limit of Spin Lifetime in Solid-State Electronic Spins. *J. Phys. Chem. Lett.* **2020**, *11*, 6273–6278.

(36) Neese, F.; Wennmohs, F.; Becker, U.; Riplinger, C. The ORCA quantum chemistry program package. *J. Chem. Phys.* **2020**, *152*, 224108.

(37) Stephens, P. J.; Devlin, F. J.; Chabalowski, C. F.; Frisch, M. J. Ab Initio Calculation of Vibrational Absorption and Circular Dichroism Spectra Using Density Functional Force Fields. *J. Phys. Chem.* **1994**, *98*, 11623–11627.

(38) Weigend, F.; Ahlrichs, R. Balanced basis sets of split valence, triple zeta valence and quadruple zeta valence quality for H to Rn: Design and assessment of accuracy. *Phys. Chem. Chem. Phys.* **2005**, *7*, 3297.

(39) Bain, G. A.; Berry, J. F. Diamagnetic Corrections and Pascal's Constants. *J. Chem. Educ.* **2008**, *85*, 532.

(40) Kurkijärvi, J. Intrinsic Fluctuations in a Superconducting Ring Closed with a Josephson Junction. *Phys. Rev. B* **1972**, *6*, 832–835.

(41) Perfetti, M. Cantilever torque magnetometry on coordination compounds: from theory to experiments. *Coord. Chem. Rev.* **2017**, *348*, 171–186.
